# Gaze processing across psychosis proneness, social anxiety, and autism traits: Examining the role of sensory quality and context congruity

**DOI:** 10.1371/journal.pone.0352116

**Published:** 2026-09-03

**Authors:** Pan Gu, Scott D. Blain, Carly A. Lasagna, Takakuni Suzuki, Cynthia Z. Burton, Katharine N. Thakkar, Jerillyn S. Kent, Ivy F. Tso

**Affiliations:** 1 Department of Psychology, School of Behavioral and Brain Sciences, The University of Texas at Dallas, Richardson, Texas, United States of America; 2 Department of Psychiatry and Behavioral Health, The Ohio State University, Columbus, Ohio, United States of America; 3 Department of Psychiatry, University of Michigan, Ann Arbor, Michigan, United States of America; 4 Department of Psychology, University of Michigan, Ann Arbor, Michigan, United States of America; 5 Department of Psychology, The University of Tulsa, Tulsa, Oklahoma, United States of America; 6 Department of Psychology, Michigan State University, East Lansing, Michigan, United States of America; University of Canberra, AUSTRALIA

## Abstract

The ability to accurately perceive gaze direction is an essential social skill but is impaired across psychopathologies exhibiting social dysfunction (e.g., schizophrenia, social anxiety disorder, autism spectrum disorder). Disrupted sensory processing and self-referential processing may both lead to aberrant gaze perception, but their respective contributions in different psychopathologies are unclear. A general population sample of 106 participants completed an online psychophysical gaze perception task and a battery of psychopathology trait questionnaires. Participants viewed face images with 9 gaze angles, superimposed with 3 levels (no, low, high) of sensory noise and in 2 (forward, deviated) head orientations, and indicated perceived self-directed gaze (yes/no) for each face. Psychophysical properties of the gaze perception curve (width, threshold) were used to index perceptual imprecision and self-referential bias, respectively. Decreased precision was associated with higher autism traits but lower social anxiety, supporting differential contributions of sensory processing among psychopathology dimensions. Subjects showed decreased precision and increased self-referential bias, when viewing stimuli with added visual noise or incongruent gaze-head directions, resembling performance previously observed in patients with schizophrenia. Additionally, higher levels of social anxiety statistically predicted a greater decrease in precision when sensory noise was added. Findings are discussed in relation to the experimental psychopathology literature and in the context of self-referential gaze perception as an adaptive default in ambiguous contexts. Overall, this work enhances our basic scientific understanding of the cognitive components that contribute to gaze perception, while advancing our knowledge of how these components are disrupted as a function of subclinical psychopathology.

## Introduction

Eye gaze is an important source of social information [1]. Perceiving and interpreting gaze direction helps us infer others’ intentions, identify the targets of their attention, and predict future communication [2]. As such, the ability to precisely and efficiently identify others’ gaze direction is critical for navigating day-to-day interactions and represents a core component of broader social cognition [3,4]. This ability, however, is altered across psychopathologies characterized by social dysfunction, including schizophrenia [5–7], autism spectrum disorder (ASD; [8–11]), social anxiety disorder [12–14], and in populations with subclinical traits of these disorders [15–17], implying that it may impact social cognition across multiple psychopathology dimensions and contribute to poorer social functioning. Therefore, disentangling the underlying components of gaze perception has the potential to elucidate the mechanisms contributing to social dysfunction. In this study, we combined experimental manipulations of stimuli in a gaze perception task and psychopathology trait measures to investigate mechanisms of altered gaze processing across psychopathology dimensions.

Gaze perception involves both early visual processing (e.g., decoding the iris/pupil position relative to the sclera) and higher-level cognition (e.g., self-referential processing). When perceiving a gaze, visual signals—such as the contrast between the iris and the sclera—are first processed through the sensory system. These sensory inputs are then integrated with stored knowledge and self-referential expectations to form a holistic representation of whether or not others are looking at me [18,19]. Aberrant gaze perception could arise from disruptions in either of these mechanisms. For example, previous work suggests that degraded visual information (e.g., poor sensory quality, low luminance, or greater viewing distance)—which disrupts low-level visual processing—can compromise healthy individuals’ precision when judging gaze direction [20–22]. In addition, judgments of gaze direction can be modulated by information derived from other cues [23]—for example, head orientation [24–27]. Research has shown that head orientation strongly biases gaze judgments [26,28,29]. When directional signals from the head conflict with encoded gaze directions (e.g., averted face with direct gaze), participants show longer reaction times [26], reduced accuracy in discriminating gaze direction [25], and stronger self-referential tendencies [12].

Altered gaze perception has not only been reported in psychosis, ASD, and social anxiety disorder [8,14,30], but also in individuals with subclinical traits related to these disorders. For instance, using the Cone of Direct Gaze (CoDG)—the range of gaze angles that one reports as receiving eye contact [21]—individuals with higher levels of social anxiety or psychosis proneness have been found to endorse a wider CoDG, suggesting they perceive direct eye contact from a wider range of gaze directions [13,17,31]. Individuals who endorse more autism traits have similarly shown altered gaze perception, including decreased sensitivity to direct gaze [15] and a greater reliance on featural information (e.g., the size of the eyes)—rather than configural information (e.g., distance between facial features)—to detect self-directed gaze [32]. These findings suggest that aberrant gaze perception is not an all-or-nothing phenomenon that appears only in clinical populations; rather, it can also be present in the general population and the degree of disruption likely scales with levels of psychopathology. Furthermore, aberrant gaze perception observed across various psychopathology dimensions may be differentially subject to alterations in early visual and higher-level cognitive processing. However, previous gaze perception studies primarily employed methods that are descriptive in nature (e.g., CoDG) and failed to distinguish underlying processes from one another. To delineate how these processes may be **similarly** and **differentially** disrupted across psychopathology dimensions, it is crucial to deploy methods that can parse disruptions in low-level visual processing from those in higher-level cognition.

In the current study, we used a psychophysical approach to decode the respective contributions of each process in the context of self-directed gaze perception. Participants briefly viewed face images and indicated whether they believed they were being looked at. By fitting a psychophysical function to participant-level eye-contact endorsement data, two critical perceptual properties—width and threshold—could be derived. Width indicates the imprecision of perceptual judgments, indexing the amount of gaze turn needed to detect a shift from the presence of to the absence of eye contact, regulated by early visual processing [33,34]. Threshold reflects the degree of deviation from direct gaze that is interpreted as self-referential half of the time. Given that participants must explicitly judge whether gaze is directed at themselves across a continuum of gaze deviations, rather than simply detect eye orientation, this parameter primarily implicates higher-order cognitive processing [6,35]. We used threshold and width to quantify two key processes and examined their relationships with three psychopathology dimensions in a general population sample: psychosis proneness, autism, and social anxiety. We hypothesized that psychopathology dimensions would show differential levels of disruptions in these two processes, such that: 1) social anxiety would be associated with greater self-referential bias, in line with the wider CoDG reported in individuals with higher social anxiety [12,13,31,36], indicating a tendency to judge a broader range of gaze directions as self-directed; 2) autism would be associated with lower perceptual precision, as individuals with autism were less precise in detecting subtle changes in gaze angle [37]; and 3) psychosis proneness would be associated with lower perceptual precision and higher self-referential bias, consistent with prior findings linking psychosis proneness to wider CoDG and reduced precision [17,33].

Additionally, we investigated whether the gaze perception of individuals with different levels of psychopathology would be differentially susceptible to changes in the sensory quality and context congruity of the stimuli; this would allow us to further infer the relative contributions of low-level visual processing and higher-order cognition to gaze perception impairments. To do so, we manipulated sensory quality and context congruity, respectively, by using face images with superimposed visual noise and in different head orientations. We anticipated that: 1) adding visual noise would decrease perceptual precision, whereas the deviated-head condition would increase self-referential bias [33]; 2) higher autism traits would be associated with less change in self-referential bias when head orientation is manipulated, as those with autism were less sensitive to social cues [15]; 3) individuals with higher psychosis proneness would display worse perceptual precision and stronger self-directed bias, in conditions with higher visual noise and deviated heads respectively, given the consistent prevalence of basic visual deficits and ideas of reference in psychosis [38,39].

## Materials and methods

### Participants

Participants from English-speaking countries were recruited via Prolific Academic (www.prolific.ac). For first timepoint (Part I), 120 participants were recruited with balanced sex and age distributions (30 per sex/age group: 18–25 and 26–30). After data quality review, the final sample of Part I included 106 participants (see exclusion criteria in supplementary; demographics in Table 1). Participants were invited back to complete the task again after two weeks (Part II), with 83 participants providing valid data at both timepoints.

**Table 1:** Demographic characteristics, self-report psychopathology measures, and psychometric parameters derived from the gaze perception task (threshold, width) of participants in Part I.

Variable	N	Mean	SD	Min	Max
Demographics					
Age (years)	106	24.7	3.9	18	30
18-25	54	21.4	2.4	18	25
26-30	52	28.2	1.4	26	30
Sex (% Female)	106	50.9			
Race/Ethnicity					
White	66				
Black	7				
Asian	16				
More than one race	4				
Other/NR	13				
Education (years)	106	15.2	2.1	12	21
Parental Education (years)	106	14.5	3	8	21
Cardiff Anomalous Perceptions Scale (CAPS)					
Total Score	106	46.5	46.9	0	226
Total number of experiences	106	5.4	5.2	0	27
Intrusiveness score	106	14.7	15.4	0	77
Frequency score	106	13.1	14.9	0	77
Distress score	106	13	14	0	73
Peters et al. Delusions Inventory (PDI)					
Total score	106	43.3	35.9	0	146
Distress scale	106	11.7	10.2	0	43
Preoccupation scale	106	12.5	11	0	50
Conviction scale	106	14.6	12.2	0	59
Social Phobia Inventory (SPIN)	106	22.9	13.6	1	63
Social Anxiety Disorder Dimensional Scale (SAD-D)	106	10.9	7.7	0	33
Autism Spectrum Quotient (AQ)					
Total score	106	115.9	14.9	78	156
Social skills scale	106	22.9	5.4	12	36
Attention switching scale	106	25.2	3.9	14	35
Attention to detail scale	106	24.7	4.1	14	37
Communication scale	106	21.9	4.7	10	35
Imagination scale	106	21.2	4.1	11	33
Gaze Perception Metrics in Angle of Gaze Aversion					
Perceptual Imprecision (Width)					
Forward No Noise	106	10.47	3.75	3.16	21.67
Deviated No Noise	106	16.05	8.32	5.60	53.32
Forward Low Noise	105	12.53	4.09	4.67	23.91
Forward High Noise	104	14.57	3.67	7.28	24.64
Self-referential Bias (Threshold)					
Forward No Noise	106	4.10	1.59	0.25	7.47
Deviated No Noise	106	10.09	2.78	3.00	18.41
Forward Low Noise	105	4.76	1.98	−0.12	8.81
Forward High Noise	104	5.66	2.28	−0.15	11.93

*Note.* Gaze perception metrics were reported in the unit of angle of gaze aversion: greater precision is reflected by smaller width, and greater self-referential beliefs is indexed by higher threshold. SD = Standard deviation; Min = Minimum value; Max = Maximum value; NR = Prefer not to disclose; Education = The years of highest completed education, including ongoing degrees; Parent education = The highest education between two parents in years.

### Procedure

The data were collected from Prolific between August 14^th^ and September 1^st^, 2020. Individuals who expressed interest in the study on Prolific were redirected to an anonymous Qualtrics survey (Provo, UT), where they were informed of study procedures before providing written consent to participation. Participants completed a demographic form, a psychophysical gaze perception task, and a battery of self-report psychopathology measures for Part I. Then, they repeated the gaze perception task two weeks later in Part II to examine test-retest reliability of the gaze perception metrics (see supplementary materials). This step is crucial for unsupervised online task administration, which is susceptible to more environmental confounds compared to supervised in-lab administration. The study was approved by and received exempt status from the Institutional Review Board at the University of Michigan Medical School, as it involved no more than minimal risk and did not include identifiable information (Study ID: HUM00184613, OHRP IRB Registration Number(s): IRB00000246).

### Gaze perception task

**Fig 1:**
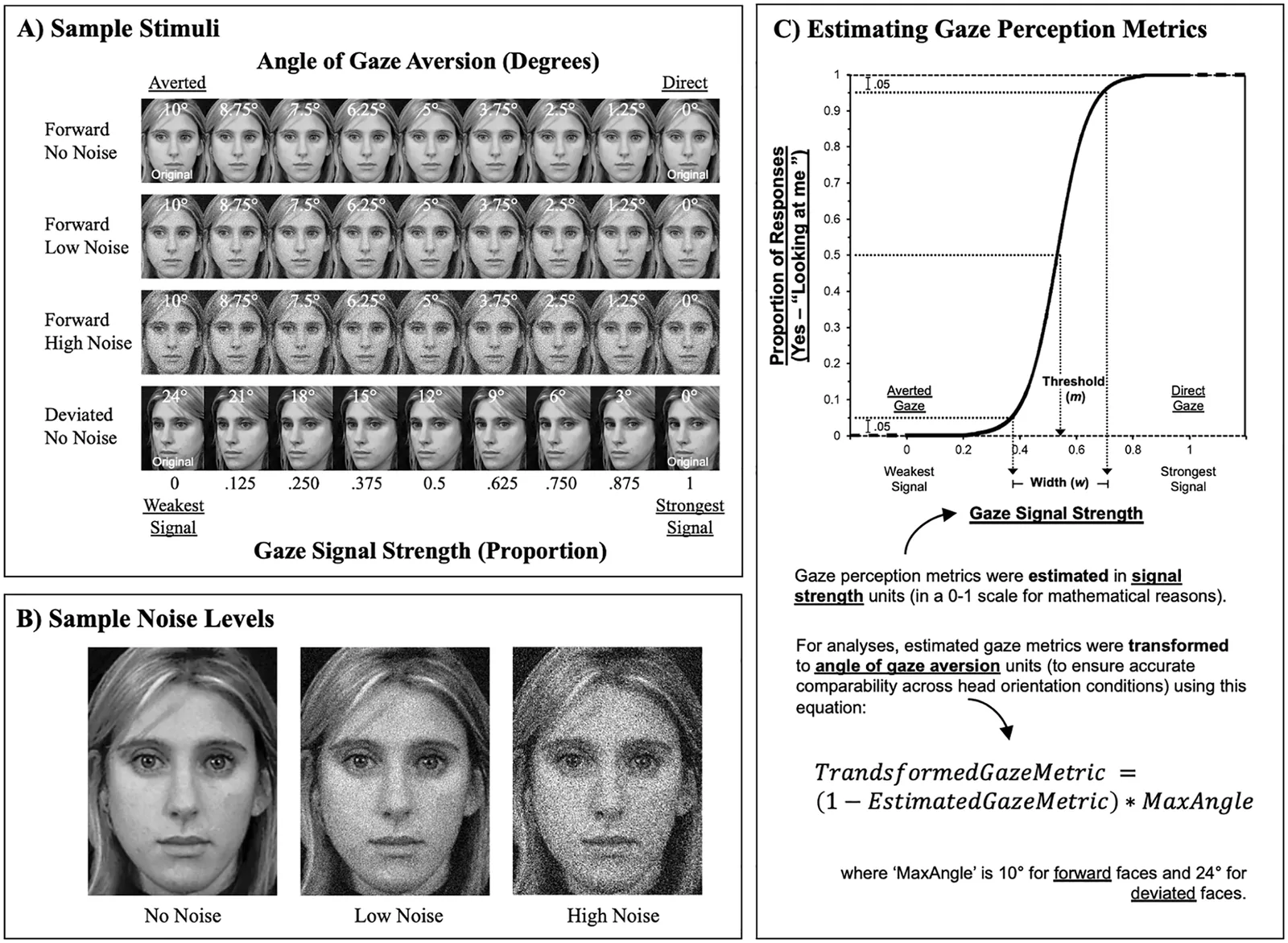
Sample stimuli and performance estimation procedures for gaze perception task. *Note.* (A) Example of stimuli for a female actor in the gaze perception task. The task used forward faces with gaze arranged from 0º to 10º and deviated faces (bottom row) with gaze arranged from 0º to 24º. The top row illustrates face stimuli without noise; the next two rows illustrate face stimuli with added levels of noise. Each condition had 108 trials, consisting of 12 stimuli presentations at each of nine gaze angles, resulting in 432 total trials (6 actors x 9 gaze angles x 4 conditions x 2 laterality). Stimuli order within each condition was pseudorandomized and held constant across participants (see supplementary materials for calibration procedure). Adapted from Fig 1 in Lasagna et al. (2020), PLOS ONE, 15(3): e0230258, under a CC BY 4.0 license (https://creativecommons.org/licenses/by/4.0/). (B) More detailed example of stimuli with different levels of superimposed gaussian noise. Adapted from Fig 1 in Lasagna et al. (2020), PLOS ONE, 15(3): e0230258, under a CC BY 4.0 license. (C) Psychometric function and parameters for gaze perception. Given the psychophysical approach, gaze angles can also be conceptualized in terms of “gaze signal strength”, where the most-self-directed gaze angle (0º) has a signal strength of 1 and the most-deviated gaze angle (i.e., 10º for forward faces; 24º for deviated faces) has a signal strength of 0. Estimation (in units of gaze signal strength from 0 to 1) yielded two parameters for each participant: threshold (*m*) and width (w). These parameters were then transformed into units of “angle of gaze aversion” for analysis using the equation shown above. After this transformation, the threshold indexes the gaze angle corresponding to 50% endorsement and reflects one’s self-referential bias. The width indexes the differences between the gaze angles at which the function reaches 5% and 95% endorsement. This indexes perceptual imprecision, or the sensitivity a participant demonstrates in detecting changes in stimulus gaze angle.

The Gaze Perception task was adapted from Lasagna et al. [33] with revised gaze angles. Participants viewed face stimuli with 9 gaze angles, superimposed with 3 levels (no, low, high) of sensory noise, and in 2 head orientations (forward, 30º deviated), and indicated whether they perceive self-directed gaze (yes/no) to each face (Fig 1). Totally four conditions were completed in the following order of increasing difficulty: (1) forward faces, no noise, (2) deviated faces, no noise, (3) forward faces, low noise, and (4) forward faces, high noise.

#### Stimuli.

Nine incremental gaze angles were created by morphing realistic grayscale images from George et al. [40], consisting of six actors (three male, three female), using the “Abrosoft Fanta Morph” software (Beijing, China; see details in supplementary materials). Stimuli consisted of six actors depicted in two head orientations, forward faces and deviated faces (with head averted left or right). For forward faces, gaze angles spanned equal increments from 0º (‘looking at me’) to 10º (‘looking way from me’; Fig 1A). Two noise conditions were created by superimposing Gaussian white noise over the original forward-facing images (low noise: [μ = 0, var = .01]; high noise: [μ = 0, var = .05]) using MATLAB’s Image Processing Toolbox (R2019a; Fig 1B). Deviated faces employed a wider range (0º-24º), as head deviation dramatically shifted the threshold of the psychometric curve to the left and results in >50% endorsement for even the most “averted” gaze angle [33]. Pilot testing showed that the increased range resulted in an approximately centered psychometric curve and thus more valid estimations of gaze metrics.

#### Estimating gaze perception metrics (threshold, width).

A logistic function was fitted to individuals’ eye-contact endorsement data using Bayesian estimation with default priors (psignfit 4 MATLAB toolbox; [41]) to estimate gaze parameters—*m* and *w*—in units of “gaze signal strength” on a 0–1 scale (for purely mathematical reasons; see Fig 1A for correspondence between angle of gaze aversion and gaze signal strength). To ensure accurate comparability across head orientations (which had different ranges of gaze angles) during statistical analyses, the parameters were then converted back to units of “angle of gaze aversion” in degrees (Fig 1C). The parameter *m* represents participants’ threshold—the degree of gaze deviation from direct that is interpreted as self-referential half of the time. This indexes *self-referential bias*, where higher values indicate stronger bias. The parameter *w* represents participants’ width of the function—the difference between gaze angles at which eye contact is endorsed 5% and 95% of the time. This indexes participants’ *perceptual imprecision*, with greater values indicating worse sensitivity to subtle changes in gaze angle. Further details were described in supplemental materials.

### Self-report psychopathology measures

#### Trait psychosis proneness.

**Cardiff Anomalous Perceptions Scale (CAPS; [42]):** The 32-item CAPS measures perceptual anomalies (e.g., “Do you hear your own thoughts repeated or echoed?”). Each item is initially presented as a yes/no question; then participants rate the endorsed perceptual experience on three 5-point Likert subscales (1–5) for distress, intrusiveness, and frequency of occurrences. Four separate scores can be derived: the total number of experiences (0–32), intrusiveness score (0–160), frequency score (0–160), and distress score (0–160); higher scores indicate greater frequency/severity. These were summed for a CAPS total score.

**Peters et al. Delusions Inventory (PDI; [43,44]):** The PDI is a 21-item questionnaire measuring delusional ideation in the general population. Participants first answered a yes/no question about a delusional belief and rated endorsed belief with three 5-point Likert subscales (1–5) for distress, preoccupation, and conviction; higher scores correspond to greater frequency/severity. A PDI total score is obtained by adding the three subscales and the yes/no scores.

#### Trait social anxiety.

**Social Phobia Inventory (SPIN; [45]):** The 17-item SPIN is designed to measure social phobia severity. The statements assessed fear, avoidance, and physiological symptoms over the past week. Participants rated each statement on a 5-point Likert scale, from 1 (“not at all bothered”) to 5 (“extremely bothered”), with higher scores corresponding to greater severity.

**Social Anxiety Disorder Dimensional Scale (SAD-D; [46]):** This 10-item scale provides dimensional ratings of categorical diagnoses included in The Diagnostic and Statistical Manual of Mental Disorders (5th ed.; [47]). Participants rated the frequency of social anxiety symptoms over the past month on a 5-point Likert scale from 0 (“never”) to 4 (“all of the time”), with higher scores indicating greater distress.

#### Autism traits.

**Autism Spectrum Quotient (AQ; [48]):** A 50-item adult autism spectrum screener assesses five domains of functioning: social skill, attention switching, attention to detail, communication, and imagination. Items were scored on a 4-point Likert-scale (from 1 = “definitely disagree” to 4 = “definitely agree”) after relevant items were reverse coded. This scoring system, relative to the original binary system (agree vs. disagree), has been shown to improve internal consistency, test-retest reliability, and total variance captured by the measure [49].

### Statistical analyses

Psychopathology trait factors were calculated using exploratory structural equation modeling (ESEM), implemented in Mplus [50]. An oblique three-factor solution was calculated for total scores on the CAPS, PDI, SAD-D, and SPIN as well as scores on the five AQ subscales scores—anticipating factors would emerge for autism traits, psychosis proneness, and social anxiety. These factors were then used to predict the gaze perception metrics (width, threshold; see model specifications in supplemental materials and S1 Fig). ESEM can accommodate interrelated psychopathology traits and nontrivial cross-loadings of indicators, offering advantages over traditional structural equation modeling with confirmatory factor analysis. Furthermore, ESEM allows more accurate model estimation, relative to conducting exploratory factor analysis and extracting factor scores for follow-up regressions [51].

Separate repeated-measures ANOVAs were conducted in IBM SPSS Statistics (Version 29) with an alpha level of *p* < .05 to determine the effects of sensory quality and context congruity, respectively, on gaze perception metrics. Huynh-Feldt epsilon correction was applied if the sphericity assumption was violated.

Subsequently, we assessed how participants’ levels of the three psychopathology factors (i.e., psychosis proneness, autism, social anxiety) were related to their susceptibility to sensory quality and context congruity manipulations. Using the slope terms (indexing a linear function of visual noise) from the ESEM analysis, we computed associations between estimated trait factors and gaze metrics across noise conditions (S1 Fig). To assess whether head orientation interacted with psychopathology dimensions to affect task performance, regression analyses were conducted using trait factor scores—derived from ESEM factor loadings—to predict head deviation change scores for gaze metrics. These change scores were computed by subtracting the no-noise condition width/threshold from deviated-head condition parameters.

## Results

### Relationship of gaze metrics with psychopathology dimensions

Our ESEM model showed good fit (*χ*2 = 96.692, *p* = .005, RMSEA = .066, 95% CI: [.037,.092], SRMR = .074, CFI = .951, TLI = .919). As anticipated, our three factors corresponded to Psychosis Proneness, Autism, and Social Anxiety (Table S2 in S1 File). Social Anxiety was positively correlated with Autism (*r* = .507, *p* < .001, 95% CI [.339,.674]) and Psychosis Proneness (*r* = .271, *p* = .003, 95% CI [.092,.451]); Autism and Psychosis Proneness were positively correlated with one another (*r* = .259, *p* = .006, 95% CI [.076,.442]). Standardized path coefficients for associations between psychopathology traits and gaze task performance are provided in Table 2.

**Table 2:** Standardized path coefficients from psychopathology traits to gaze perception task performance.

Criterion Variable	*β*	95% CI	*p*
Perceptual Imprecision_Intercept_			
Psychosis Proneness	−.012	[-.251,.228]	.922
Autism	.408	[.027,.790]	.036*
Social Anxiety	−.469	[-.814, -.124]	.008*
Perceptual Impercision_Slope_			
Psychosis Proneness	−.094	[-.359,.171]	.485
Autism	−.327	[-.956,.303]	.309
Social Anxiety	.582	[.021, 1.143]	.042*
Self-referential Bias_Intercept_			
Psychosis Proneness	−.117	[-.367,.132]	.357
Autism	.305	[.009,.600]	.044*
Social Anxiety	−.200	[-.531,.130]	.234
Self-referential Bias_Slope_			
Psychosis Proneness	−.052	[-.274,.171]	.649
Autism	.072	[-.208,.352]	.615
Social Anxiety	−.062	[-.379,.254]	.700

*Note.* *p < .05.

Social Anxiety was negatively associated with Perceptual Imprecision_Intercept_, suggesting more precise gaze perception among those with higher social anxiety (Table 2). In contrast, Autism was positively associated with Perceptual Imprecision_Intercept_, suggesting lower precision among those with higher autism traits. Higher Self-referential Bias_Intercept_ was correlated with higher autism traits. Models incorporating observations from Part II are reported in our supplemental materials.

### Sensory quality effects on gaze processing

**Fig 2:**
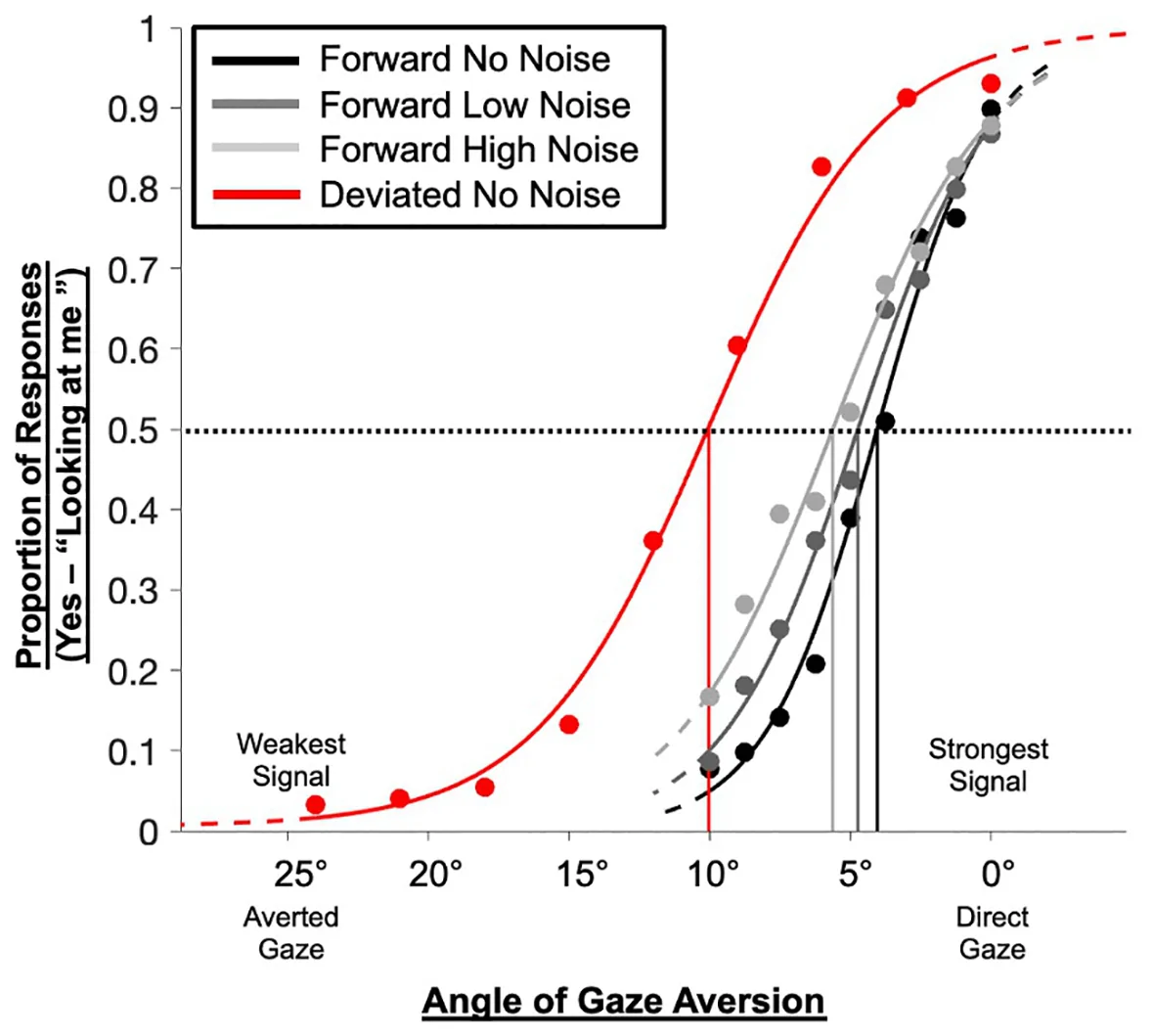
Fitted curves of eye-contact endorsement rates across sensory quality and context congruity. *Note.* The mean proportion of ‘Yes-looking at me’ responses for each angle of gaze aversion was plotted across two head orientations (forward faces, deviated faces) and different noise levels for forward faces only (no noise, low noise, high noise). The vertical lines index the 50% threshold (self-referential bias) for each condition.

Repeated-measures ANOVA showed that perceptual imprecision (width) differed significantly across noise levels, *F*(1.892, 191.123) = 66.126, *MSE* = 7.412, *p* < .001, such that participants showed high imprecision (increased width) as sensory noise increased (Fig 2). Follow-up comparisons with Bonferroni adjustments using local error terms (ESCI software; [52]) indicated increased imprecision from no to low-noise levels (*d*_*unb*_ = 0.515, 95% CI: [0.336, 0.700]) and from low- to high-noise levels (*d*_*unb*_ = 0.599, 95% CI: [0.404, 0.801]). Self-referential bias (threshold) also increased significantly with noise (*F*(1.885, 190.398) = 61.475, *MSE* = 1.097, *p* < .001), showing stronger bias (higher threshold with increased avertedness of gaze direction) from no- to low-noise levels (*d*_*unb*_ = 0.354, 95% CI: [0.192, 0.518]) and from low- to high-noise levels (*d*_*unb*_ = 0.404, 95% CI: [0.267, 0.545]).

### Context congruity effects on gaze processing

Significant effects were found on perceptual imprecision (width; *F*(1,104) = 63.469, *MSE* = 26.006, *p* < .001) and self-referential bias (threshold; *F*(1,104) = 677.484, *MSE* = 2.806, *p* < .001) between head orientations. In general, participants had lower precision when processing deviated faces compared to forward faces, and they interpreted further averted gaze as self-directed when the head was deviated.

### Interactions between psychopathology traits and manipulations of gaze processing

**Fig 3:**
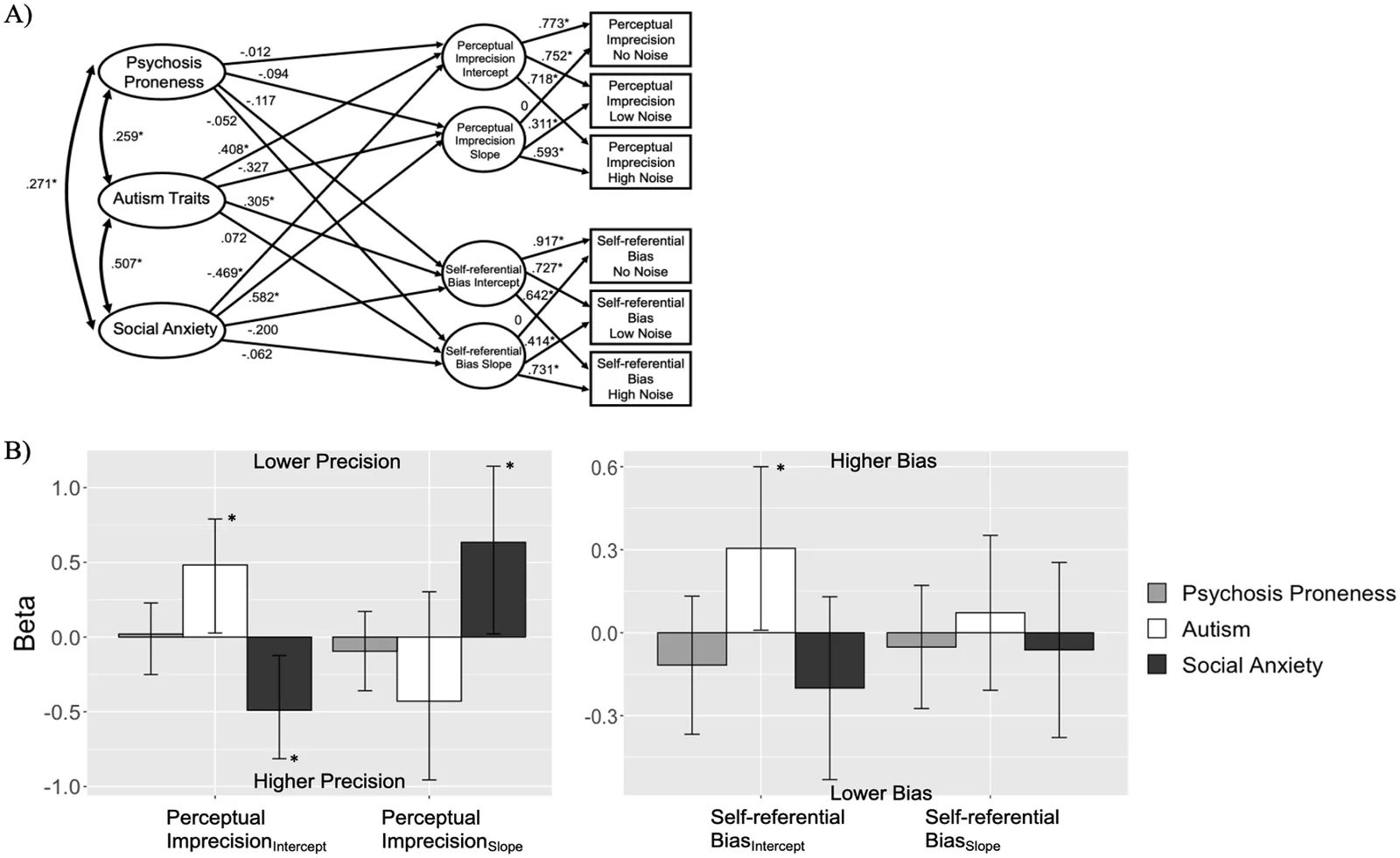
Standard path coefficients of perceptual imprecision and self-referential bias in ESEM model. *Note.* (A) Standardized path coefficients are shown between psychopathology traits and gaze perception metrics in the left portion. On the right, each of the perceptual imprecision (width) and self-referential bias (threshold) variables showed significant loadings onto the corresponding intercept latent variables (task performance irrespective of visual noise). (B) Standardized path coefficients are shown (with 95% confidence intervals) for the associations of psychopathology traits with gaze perception metrics intercept (irrespective of noise) and slope (impact of visual noise on parameters).

Participants with higher social anxiety exhibited a stronger effect of noise on imprecision, such that their imprecision increased with increasing levels of noise (Fig 3). In general, the associations between psychopathology traits and gaze metrics were not influenced by head orientation (Table S3 in S1 File).

## Discussion

The current study combined a psychophysical gaze perception task with experimental stimulus manipulations, self-reports of psychopathology traits, and structural equation modeling to examine components of gaze perception and their disruptions. Experimental manipulations of sensory quality (i.e., introducing gaussian noise over the stimuli) and context congruity (i.e., deviated-head orientation) successfully reduced perceptual precision and increased self-referential bias, respectively. Supporting our overarching hypothesis, the findings revealed that distinct psychopathology dimensions were associated with differential disruptions in gaze perception: autism traits were associated with lower perceptual precision, while higher social anxiety was associated with greater precision. However, not all predicted associations were supported, and the directions of some effects differed from our initial expectations. Specific hypotheses and interpretations are discussed below, organized first around the basic experimental effects and then around their associations with psychopathology traits.

This study first examined the impact of experimental stimulus manipulations on underlying components of gaze perception. As expected, low-level sensory manipulations (i.e., adding visual noise) led to reduced perceptual precision, consistent with previous work [20,21,53]. The visual system receives less relevant information when a stimulus has poorer sensory quality. Consequently, participants’ decoding of gaze signals was likely impeded and their precision for judging gaze direction decreased [54,55]. We did not hypothesize that higher-level cognitive manipulations (i.e., the deviated-head condition) would reduce precision, but this is also consistent with prior research [2,12]. This may emerge from incongruity between information sources (e.g., eyes and head). Although gaze can signal an individual’s target of attention, observers tend to perceive the face as a whole and exploit multiple sources of information when making judgments [23,56]. When context incongruity arises, it may disrupt participants’ certainty about their judgments, resulting in decreased precision. The repulsive effect of head orientation may further contribute to this pattern, whereby a head rotated 30º to the left biases perceived gaze direction rightward (e.g., a true gaze of 25º leftward may be perceived as ~20º leftward) [21,57,58]. As a result, slightly averted gazes may be more likely to be judged as “looking at me”, whereas truly direct gaze may be perceived as slightly averted, thereby reducing precision.

Unexpectedly, both stimulus manipulations also led to increased self-referential bias. Though not hypothesized, similar effects have been found for manipulations of visual noise [59,60] and head orientation [33]. Mareschal et al. [60] proposed that—when gaze signal is ambiguous—observers may revert to a ‘default’ belief that gaze is self-directed. This is consistent with Bayesian decision frameworks, which posits that individuals rely heavily on prior expectations to compensate for unreliable inputs [61]. In our study, both low-level sensory degradation and higher-level context incongruity likely introduced unreliable inputs, causing participants to default to prior beliefs favoring direct gaze [60]. This bias may have adaptive qualities—as direct gaze can often signal a potential threat or positive attention (e.g., desire to bond). Failure to detect these cues might result in negative consequences, and thus, the consequences of missing true direct gaze (e.g., ignoring a stranger’s stare) likely outweigh the consequences of false alarms (e.g., embarrassment from waving at someone who wasn’t looking at you; [62]).

Our second aim discerned whether psychopathology dimensions are differentially associated with—and susceptible to disruptions in—gaze perception components. The distributions of psychopathology traits in our sample were broadly consistent with those reported in larger non-clinical samples, with mean levels of autism [49,63] and social anxiety [45,64,65] traits being comparably a bit higher, and  mean psychosis proneness being slightly lower [66]. Autism traits were associated with *lower precision* as hypothesized; this aligns with worse precision exhibited in ASD when discriminating subtle changes in gaze angle [8,37,67]. This pattern may reflect a broader perceptual uncertainty in individuals with higher autism traits, rather than reduced attention to the eye region. While ASD and higher autism traits are often associated with gaze avoidance, recent eye-tracking evidence from a transdiagnostic sample (including participants spanning from neurotypical to subclinical autism traits to ASD) using the same gaze perception task [68] indicated that the relationship between autism traits and reduced dwell time on the eyes emerges only after gaze direction judgements were made. This argues against a simple attentional account for worse precision among those high in autism traits. Subjects with more autism traits also exhibited *higher self-referential bias*, suggesting they overestimated averted gaze as self-directed. Though this finding contradicts the result of Matsuyoshi et al. [32]—autism traits negatively correlated with self-referential bias—Dratsch et al. [69] suggested such inconsistencies may emerge from differences in the range of gaze angles examined. Elevated self-referential bias in ASD was specific to subtly averted gaze angles (1º-7º), whereas their performance was similar to controls for broader gaze-angle ranges (15º-25º; [69]). Further support is found in studies that examined gaze perception with similarly stringent ranges (e.g., 0º-15º in [61]; 0º-10º in [70]). Taken together, these findings dovetail with the hypo-prior accounts of perceptual experience in ASD [71], which postulates that weaker or less precise priors lead to a reduced capacity to resolve ambiguity through top-down processes, resulting in increased uncertainty and downstream biases in social inference. Future studies are needed to examine whether these findings generalize to individuals with a clinical diagnosis of ASD.

Converse to autism traits, subjects with higher social anxiety unexpectedly showed *greater precision* when perceiving changes in gaze angle. This coincides with a growing body of work linking anxiety to *enhanced* social cognitive abilities [72–74]. Enhanced performance among those with higher social anxiety may reflect increased attention toward the task or arise from an increased sensitivity toward sensory, interoceptive, and affective information that contributes to social perception abilities [75–77]. Despite positive associations with overall precision, social anxiety was related to stronger negative effects of sensory noise on precision. In other words, although subjects with high social anxiety initially showed superior precision, this was attenuated after degrading the stimuli’s sensory quality. This pattern may reflect dynamic attentional biases in anxious individuals that shift as a function of perceived threat value [78,79]. Heightened vigilance enabled them to closely monitor gaze cues, resulting in superior precision. However, when sensory noise increased ambiguity and task difficulty, socially anxious individuals may have interpreted the degraded stimuli as more threatening [80,81], instigating attentional avoidance that impeded perceptual precision [79]. More speculatively, this connection between social anxiety and degraded precision could involve the vital neurochemical role that GABA plays in anxiety and noise-suppression within the visual system [82,83]. The hypothesized relation of social anxiety to higher self-referential bias was not found and seemingly contradicts previous findings in both the general-population and clinical samples [13,14,16]. However, larger samples and/or more severe levels of social anxiety may be required to reliably detect such an effect.

Against our hypotheses, psychosis proneness was not associated with low precision or high self-referential bias, contrasting with Chan et al.‘s [84] findings of a positive association between psychosis symptom severity and self-referential bias in clinical samples. Nonetheless, our null results may relate to the limited expressions of psychopathology in this relatively healthy sample—especially for psychosis proneness (Table 1 and S3 Fig)— and the non-linear nature of this relationship across the psychosis continuum. Moreover, given findings and theories that *negative symptoms* and related traits (i.e., detachment) may be particularly connected to alterations in social cognition [72,85]—whereas the CAPS and PDI are conceptually related to *positive symptoms*—follow-up research should incorporate dimensional measures of social anhedonia and intimacy avoidance.

Finally, these findings have clinical relevance. One inspiration for the study was to examine whether experimental stimulus manipulations could induce gaze perception patterns similar to those observed in schizophrenia. Patients with schizophrenia experience greater *internal* sensory noise and they show reduced perceptual precision and enhanced self-referential bias when perceiving gaze [86]—mirroring patterns exhibited by our general-population participants following the introduction of *external* noise through stimulus manipulations. Given advances in the visual remediation literature, it is possible that teaching patients to ignore or suppress external sensory noise could lead to reductions in internal noise and therefore improved precision [87–89]. Nonetheless, direct translational applications of our study are modest, and more attention is needed for external sensory/contextual manipulations.

### Limitations and future research

A few limitations are worth noting. First, our non-clinical sample reported a restricted range of psychopathology, particularly for psychosis proneness, which may have limited observable relations with gaze perception. The relatively modest sample size may also have affected the stability of parameter estimates and reduced statistical power. Future studies should recruit large transdiagnostic samples of patients with pronounced social deficits and those with subclinical levels of traits, as proposed by Tso et al. [90]. This could further validate these relations and potentially reveal both transdiagnostic and disorder-specific treatment targets for gaze processing deficits and associated social dysfunction. Furthermore, participants completed the study during the COVID-19 pandemic, a period associated with elevated psychological stress. Although psychopathology trait scores in this sample were broadly comparable to those reported in pre-COVID studies conducted in the general population, it is possible that pandemic-related stressors may have heightened the levels of self-reported psychopathology traits [91], particularly for social anxiety [92] and autism traits [49,63]. Second, present findings suggest that adding visual noise with a fixed order design to gaze stimuli reduced perceptual precision and increased self-referential bias, yet conclusions about the impact on specific cognitive processes remain limited. Decreased perceptual precision could reflect disrupted early visual processing or changes in social cognition-specific processes. Likewise, enhanced self-referential bias could be caused by stronger reliance on prior beliefs or by strengthened perceptual biases [6]. Future work needs to carefully rule out the practice or fatigue effects and better distinguish among these alternative explanations through computational modeling and/or neuroimaging. Third, given the forced yes/no experimental design, the upper and lower asymptotes (i.e., lapse and guess rates) of the fitted logistic function were fixed when estimating gaze perception metrics. Future studies should consider occasional response errors and individual differences in subjective criteria for perceived self-directed gaze, particularly under noisier conditions, by allowing these asymptotes to vary and evaluating alternative lapse and guess rate specifications. Notwithstanding these limitations, the paper is the first to use an individual-differences approach to systematically compare shared and distinct mechanisms in gaze perception, and their associations with psychosis proneness, social anxiety, and autism traits in a community sample. This individual-differences approach has the benefit of investigating these clinical and behavioral phenotypes dimensionally, which is in line with empirical data supporting the dimensional conceptualization of psychopathology.

## Supporting information

S1 Fig Full model specification for SEM model. The left portion of the figure shows three exploratory factors derived from the nine self-report variables. The right portion of the figure shows gaze perception task performance variables, which were estimated as intercept and slope terms for perceptual imprecision (width) and self-referential bias (threshold) parameters across task conditions. Intercept terms capture task performance irrespective of sensory noise, and slope terms capture the impact of visual noise on each gaze metric. Residual variances of observed performance variables from the same task condition were allowed to correlate. Paths in the center of the diagram represent regressions of each of the four task performance latent variables onto the three self-report factors. PDI = Peters et al. Delusions Inventory, CAPS = Cardiff Anomalous Perceptions Scale, SAD-D = Social Anxiety Disorder Dimensional Scale, SPIN = Social Phobia Inventory, AQ = Autism Spectrum Quotient.
(TIF)

S2 Fig Standardized measurement model for task performance intercept and slope parameters. Each of the observed perceptual imprecision (width) and self-referential bias (threshold) variables showed significant loadings onto corresponding intercept and slope terms. Correlations between the residual variances of task performance parameters from corresponding conditions were indicated on the right.
(TIF)

S3 Fig The distribution of self-report psychopathology scores in the final sample of Part I. CAPS = Cardiff Anomalous Perceptions Scale, PDI-21 = Peters et al. Delusions Inventory, SAD-D = Social Anxiety Disorder Dimensional Scale, SPIN = Social Phobia Inventory, AQ = Autism Spectrum Quotient.
(TIF)

S1 File Methodology for gaze perception task and structural equation modeling. (DOCX)
