# Supplementary material for: Gaze processing across psychosis proneness, social anxiety, and autism traits: Examining the role of sensory quality and context congruity
Source: PLoS One. 2026 Sep 3;21(9):e0352116. doi: 10.1371/journal.pone.0352116 (PMC13541124; doi:10.1371/journal.pone.0352116)
Supplement: Supplementary file 1 [file pone.0352116.s004.docx]

**Gaze Processing Across Psychosis Proneness, Social Anxiety, and Autism Traits: Examining the Role of Sensory Quality and Context Congruity**

**Table S1.** Descriptive Statistics on the Goodness of Fit for the Final Participants’ Gaze Perception Metrics in Part I.

|  | Deviance Residual | |
| --- | --- | --- |
|  | N | M(SD) |
| Forward No Noise | 106 | 6.752(3.379) |
| Deviated No Noise | 106 | 6.996(4.362) |
| Forward Low Noise | 105 | 7.032(3.511) |
| Forward High Noise | 104 | 7.500(3.502) |
|  |  |  |

*Note.* A higher deviance residual indicates a poorer fit between the logistic function and eye contact endorsement data.

**Table S2.** Standardized Factor Loadings of Self-Report Psychopathology Measures on Three Exploratory Factors.

| Scale | Psychosis Proneness | Social Anxiety | Autism Traits |
| --- | --- | --- | --- |
| CAPS | **.973*** | -.049 | .036 |
| PDI | **.781*** | .118 | -.052 |
| SAD-D | .153 | **.635*** | .123 |
| SPIN | -.009 | **.966*** | -.003 |
| AQ – Social Skills | -.079 | .335* | **.589*** |
| AQ – Communication | .034 | -.006 | **.929*** |
| AQ – Imagination | -0.006 | -.205 | **.622*** |
| AQ – Attention Switching | .052 | .168 | **.538*** |
| AQ – Attention to Detail | **.316*** | -.006 | -.107 |
| *Note.* **p* < .05 (based on the z-distribution and standard errors computed using the Huber-White sandwich estimator); bolded values indicate which factor the scale had the largest loading for. CAPS = Cardiff Anomalous Perceptions Scale, PDI = Peters et al. Delusions Inventory, SAD-D = Social Anxiety Disorder Dimensional Scale, SPIN = Social Phobia Inventory, AQ = Autism Spectrum Quotient. | | | |

**Table S3**. Standardized Regression Coefficients from Psychopathology Factors to Gaze Perception Task Performance with Different Context Congruity.

| Criterion Variable | *Β* [95% CI] | *p* |
| --- | --- | --- |
| Head Deviation Change in Width |  |  |
| Psychosis Proneness | -.074 [-.269, .126] | .475 |
| Autism | .191 [-.049, .426] | .118 |
| Social Anxiety | -.043 [-.280, .195] | .724 |
| Head Deviation Change in Threshold |  |  |
| Psychosis Proneness | .080 [-.121, .275] | .441 |
| Autism | .071 [-.168, .307] | .562 |
| Social Anxiety | -.193 [-.428, .048] | .116 |

*Note.* **p* < .05

**Supporting Information S1.**

**1.1 Exclusion criteria for participants**

Participants from English-speaking countries were recruited via Prolific Academic ([www.prolific.ac](http://www.prolific.ac)), and individuals who expressed interest were redirected to an anonymous Qualtrics survey (Provo, UT). They were informed of study procedures before consenting to participation.

Participants were instructed to complete the survey using a desktop or laptop (no phones or tablets) in a single session and in a private environment with minimal distraction. Thirty subjects were recruited for each sex (male and female) and age group (18-25 and 26-30), for a total target sample of 120 participants. Due to a technical error, two additional participants completed the study (*n* = 1 male in the 18-25 group and *n* = 1 male in the 26-30 group), resulting in a total of 122 participants in Part I. Sixteen participants were excluded because of: failed attention checks (*n* = 5), random responses on the gaze perception task (*n* = 2), or completing the study on a smartphone or tablet (*n* = 9). In Part II, ten of the returned participants were excluded due to screen resolution mismatch with Part I (*n* = 7) or failed attention check (*n* = 3). Participants received $10 upon completion of Part I, which took an average of 45-60 minutes, and $5 for finishing Part II, which lasted for 15-20 minutes.

**1.2 Calibration procedure for gaze perception task**

Before the gaze perception task, participants followed a novel pre-task calibration procedure to adjust their browser to a standard size to better control the visual angle of task stimuli during online experimentation. Meta information, such as browser type, browser resolution, and operating system, was collected to verify that participants had completed the survey on approved devices and used the same device for Part I and Part II. To control the size of stimuli, participants were instructed to locate a credit card—an object found in most households with a globally standardized size—and resize their browser (by zooming in or out) until a credit card image on the screen matched their physical credit card. This ensured that the task stimuli would be approximately 14.5cm tall and 10.8cm wide on the screen, regardless of the physical size of the monitor being used. Next, participants were told to position themselves at eye level with the center of the screen and to sit at an arm’s length away from the screen (Brascamp, 2021). Participants viewed face stimuli and indicated if they felt each face was looking at them or not via button presses (‘Yes’ = press ‘N’; ‘No’ = press ‘M’), without a time limit.

**1.3 Morphing Face Images for Stimuli**

The original images from Geroge et al. (2001) were comprised of realistic grayscale photographs of six actors depicting two gaze directions: direct eye contact (0º; actor looking straight into the camera,) and 30º averted eye contact (actor looking 30º to the right of the camera). These photographs were also taken in two head orientations (forward, 30º deviated). To generate gaze stimuli spanning a continuous range of gaze angles, we followed the morphing protocol described in our previous work (Lasagna et al., 2020). In that study, 9 intermediate images per actor were created between the two original images (0º and 30º) using 10% morph increments (“Abrosoft Fanta Morph” software; Beijing, China), yielding a set of 11 images with eyes averted leftward for 0º, 3º, 6º, 9º, 12º, 15º, 18º, 21º, 24º, 27º, and 30º. Each image was horizontally mirrored to produce a matched image of rightward-averted gaze stimuli. For the present study, we selected a subset of these morphed images to create nine stimuli spanning 0º (“looking at me”) to 24º (“looking away from me”) in 3º increments for deviated faces (see Fig 1). The same process was applied to the forward-facing images, generating stimuli spanning 0º to 10º of gaze aversion for all actors.

**1.4 Estimating Gaze Perception Metrics (Threshold, Width)**

Psignifit utilizes a 5-parameter psychometric function by default (estimating: threshold *m*, width *w*, lapse rate λ, guess rate γ, and scaling factor η) to account for overdispersion caused by guessing and attentional lapses. However, because a response was required on every trial in our task and response time was unlimited, we fixed the lapse rate, guess rate, and scaling factor at 0. This eliminates the need to rescale the estimates, thus we fixed the scaling factor. We estimated two free parameters—*m* and *w*—using Bayesian inference with default priors for both *m* and *w*.

$$S\left( x \right|m,w)= \frac{1}{1+e^{-2\log\left( \frac{1}{.05} - 1 \right) \frac{x-m}{w}}}$$

The parameter *w* represents the width of the function, an inversion of slope for the psychometric function, which gauges the difference between gaze angles at which eye contact is endorsed 5% and 95% of the time. Compared to the slope parameter used in previous studies (e.g., in Lasagna et al., 2020), width has the practical advantages of being expressed in the same units as the threshold, making it easier to interpret. Furthermore, the width parameter holds consistent meaning across the family of sigmoid functions, facilitating easier comparisons across studies (Schütt et al., 2016).

To compare across different ranges of gaze angle (0º-10º for forward faces; 0º-24º for deviated faces), we converted these parameters back to units of “angle of gaze aversion” in degrees (see Figure 1C for estimation and conversion overview). This conversion was done as follows: $TransformedMetric=(1-EstimatedMetric)*MaxAngle$, where ‘MaxAngle’ was 10º for forward faces and 24º for deviated faces.

Goodness of fit was assessed by visual inspection of each participant’s fitted curves and their deviance, measured by the deviance residual (see Table S1 for descriptive statistics). Two participants had poor fitting in forward faces, high noise condition in Part I, and another participant had poor fitting in all forward face conditions in Part II. These participants were removed from subsequent analyses.

**1.5 Exploratory Structural Equation Modeling Process for Self-Report Questionnaires**

First, the total scores on the CAPS, PDI, SAD-D, and SPIN as well as scores on the five AQ subscales were entered into a parallel analysis to determine how many factors to extract (the standardized measurement model for task performance variables is displayed in Figure S2). Parallel analysis indicated that three factors were empirically suggested in the data. Thus, we used three exploratory factors derived from the nine self-report variables as endogenous latent variables in a broader ESEM model predicting task performance. Our exogenous latent variables were estimated as intercept and slope terms comprising perceptual imprecision (width) and self-referential bias (threshold) parameters across the noise conditions. Unstandardized loadings of the observed perceptual imprecision parameters—across the three task conditions including no noise, low noise, and high noise—onto the Perceptual Imprecision_intercept_ latent variable were set to 1.0. Likewise, unstandardized loadings of the three observed self-referential bias parameters onto the Self-referential Bias_intercept_ latent variable were set to 1.0. Unstandardized loadings of performance variables—from no noise, low noise, and high noise conditions—onto either the Perceptual Imprecision_slope_ or Self-referential Bias_slope_ latent variables were set to 0.0, 1.0, and 2.0, with larger loadings corresponding to task conditions with greater sensory noise. Residual variances of observed performance variables from the same task condition were allowed to correlate (e.g., perceptual imprecision and self-referential bias from the no noise condition).

Robust maximum likelihood estimation was used, and Satorra-Bentler (2001) adjusted fit indices were computed. 95% confidence intervals were estimated for path coefficients from the three psychopathology factors to the task performance latent variables (Huber, 1967; Muthén & Muthén, 2017; Satorra & Bentler, 2001; White, 1980).

Sub-scales for the AQ were included given the documented heterogeneity of the broader AQ construct. Models including sub-scales for the CAPS and PDI were also computed, but these did not provide factor loading estimates consistent with our theoretical conceptualization of latent psychological factors in the current variables (i.e., two separate CAPS and PDI factors emerged, whereas AQ and social anxiety loaded onto a single factor). This was likely due to a high degree of linear dependence among CAPS and PDI sub-scales compared to the AQ (as the number of total items participants complete on the CAPS and PDI varies directly with their score on a specific sub-scale). Attempts to resolve this issue using correlated residuals or method factors negatively impacted model fit and/or led to models that failed to converge. Thus, in our final models, total scores were used for CAPS and PDI, whereas sub-scales were used for the AQ (the SAD-D and SPIN do not have sub-scales).

To examine whether these associations held when using data from additional observations, we recalculated our model using the gaze perception parameters from participants who completed Part II. Associations with variables from Part II were not significant, likely due to the smaller sample size available (*N* = 83).

**1.6 Test-retest Reliability of Gaze Perception Metrics**

Test-retest reliability of gaze perception metrics for each visual noise condition was calculated using intra-class correlation (ICC) coefficients—based on absolute agreement—for participants who completed both parts of the study. For self-referential bias (threshold), strong test-retest reliability was found for all noise conditions: ICC = .861 for no noise (*N* = 82), ICC = .810 for low noise (*N* = 81), and ICC = .881 for high noise (*N* = 80). Test-retest reliability was fair to moderate for perceptual imprecision (width) with ICC = .856, .770, .544 for no noise, low noise, and high noise conditions, respectively. A similar level of reliability of self-referential bias (ICC = .815, N =83) and perceptual imprecision (ICC = .799, N = 83) was seen for deviated faces. Notably, perceptual imprecision and self-referential bias demonstrated excellent test-retest reliability for the no noise condition regardless of head orientations. It is worth briefly commenting on the temporal stability of our two gaze perception components. In line with our previous work (Lasagna et al., 2020), self-referential bias exhibited strong test-retest reliability. It is particularly remarkable that self-referential bias remained temporally stable when sensory noise was added, whereas perceptual precision did not; this immunity to noise aligns well with our top-down conceptualization of self-referential bias. Notably, the width parameter used to index perceptual imprecision in the current study showed higher test-retest reliability compared to the similar slope parameter used in our previous work (Lasagna et al., 2020), which furthers prevailing arguments that favor width (versus slope) parameterizations of psychophysical logistic curves (Wichmann & Jäkel, 2018).

**References**

Brascamp, J. W. (2021). Controlling the spatial dimensions of visual stimuli in online experiments. *Journal of Vision*, *21*(8), 19. https://doi.org/10.1167/jov.21.8.19

George, N., Driver, J., & Dolan, R. J. (2001). Seen gaze-direction modulates fusiform activity and its coupling with other brain areas during face processing. *NeuroImage*, *13*(6), 1102–1112. https://doi.org/10.1006/nimg.2001.0769

Huber, P. J. (1967). The behavior of maximum likelihood estimates under nonstandard conditions. *Proceedings of the Fifth Berkeley Symposium on Mathematical Statistics and Probability*, *1: Statist*(1), 221–223.

Lasagna, C. A., McLaughlin, M. M., Deng, W. Y., Whiting, E. L., & Tso, I. F. (2020). Deconstructing eye contact perception: Measuring perceptual precision and self-referential tendency using an online psychophysical eye contact detection task. *PLoS ONE*, *15*(3). https://doi.org/10.1371/journal.pone.0230258

Muthén, L. K., & Muthén, B. O. (2017). *Mplus user’s guide.* (Eighth Edi). Los Angeles, CA.

Satorra, A., & Bentler, P. M. (2001). A scaled difference chi-square test statistic for moment structure analysis. *Psychometrika*, *66*(4), 507–514. https://doi.org/10.1007/BF02296192

Schütt, H. H., Harmeling, S., Macke, J. H., & Wichmann, F. A. (2016). Painfree and accurate Bayesian estimation of psychometric functions for (potentially) overdispersed data. *Vision Research*, *122*, 105–123. https://doi.org/10.1016/j.visres.2016.02.002

White, H. (1980). A heteroskedasticity-consistent covariance matrix estimator and a direct test for heteroskedasticity. *Econometrica*, *48*(4), 817–838. https://doi.org/10.2307/1912934

Wichmann, F. A., & Jäkel, F. (2018). Methods in Psychophysics. In J. T. Wixted (Ed.), *Stevens’ Handbook of Experimental Psychology and Cognitive Neuroscience* (1st ed., pp. 1–42). Wiley. https://doi.org/10.1002/9781119170174.epcn507
