# Supplementary material for: Gaze processing across psychosis proneness, social anxiety, and autism traits: Examining the role of sensory quality and context congruity
Source: PLoS One. 2026 Sep 3;21(9):e0352116. doi: 10.1371/journal.pone.0352116 (PMC13541124; doi:10.1371/journal.pone.0352116)
Supplement: Supplementary file 3 [file pone.0352116.s006.docx]

Dear Dr. Jeroen J. A. van Boxtel,

Thank you very much for the opportunity to submit a revision of our manuscript, “Gaze Processing Across Psychosis Proneness, Social Anxiety, and Autism Traits: Examining the Role of Sensory Quality and Context Congruity.” We also thank the anonymous reviewers for their time, thoughtful comments, and guidance to improve the original submission. We have carefully revised the manuscript to address the noted concerns and incorporate the suggested revisions. Importantly, the de-identified dataset is now publicly available on OSF: <https://osf.io/chqw2>. We believe the manuscript has substantially improved. Below, we detail our responses to each of the reviewer’s comments (which are in bold). We sometimes repeat our responses for similar comments raised by multiple reviewers for ease of review. Thank you very much for your valuable time and effort in improving this manuscript.

Sincerely,

Pan Gu and co-authors

**Editor’s Notes:**

**Reviewer 1: Comments to authors:**

**Data for the study is not available. My understanding is that this is a requirement for PlosOne. I'm not in a position to comment on the robustness of the statistical approach as I'm not familiar with ESEM, but I do have some questions regarding whether the sample size is sufficient for the individual-differences analyses conducted here. Further comments on the Introduction and the Discussion are uploaded as an attachment.**

Thank you for raising this data availability concern. In response to this and the editor’s request, we have shared the dataset on OSF: <https://osf.io/chqw2>.

Regarding the sample size, we acknowledge the limitations of a relatively small sample size, and future studies are needed to replicate the current study in a bigger sample. We agree that larger samples are more powered for individual-difference analyses using SEM. However, we note that similar sample sizes have been used successfully in previous studies. For example, Schmidt, Mueller, and Roder (2011) conducted SEM to examine how social cognition mediates the relationship between neurocognitive performance and functional status among 148 schizophrenia patients.

We have also revised and expanded the Limitation section to more thoroughly address this concern, “A few limitations are worth noting. First, our non-clinical sample reported a restricted range of psychopathology, particularly for psychosis proneness, which may have limited observable relations with gaze perception. *The relatively modest sample size may also have affected the stability of parameter estimates and reduced statistical power. Future studies should recruit large transdiagnostic samples of patients with pronounced social deficits and those with subclinical levels of traits, as proposed by Tso et al. [88]. This could further validate these relations and potentially reveal both transdiagnostic and disorder-specific treatment targets for gaze processing deficits and associated social dysfunction.*”

Despite the limitation of a small sample size, this paper is the first to use an individual difference approach to systematically compare the shared and distinct mechanisms in gaze perception, and their associations with psychosis proneness, social anxiety, and autism traits in a community sample.

Citations: Schmidt, S. J., Mueller, D. R., & Roder, V. (2011). Social Cognition as a Mediator Variable Between Neurocognition and Functional Outcome in Schizophrenia: Empirical Review and New Results by Structural Equation Modeling. Schizophrenia Bulletin, 37(suppl_2), S41–S54. https://doi.org/10.1093/schbul/sbr079

**I think the Introduction and Discussion need major revisions. Introduction pg3 paragraph 2 – what constitutes prior information in the context of gaze perception not clear.**

We thank the reviewer for these valuable suggestions. We have carefully revised the manuscript to address the noted concerns regarding the introduction and discussion.

For Introduction paragraph 2 (Page 3), we thank the reviewer for raising this concern regarding the clarity of prior information in the context of gaze perception. Here, examples for higher-level cognition processes include self-referential processing, emotional expression, and attentional control. These top-down processes are particularly critical when co-present directional signals conflict with one another, such as when head orientation is incongruent with eye direction, or when pointing gestures are incongruent with eye direction (Langton, 2000). In such cases, observers draw on prior knowledge and expectations to resolve the ambiguous sources of information, form a holistic judgement of eye gaze, and most often to think others were “looking at me” (Mareschal et al., 2013). We have revised the paragraph to reflect these changes. Specifically, on page 3, we now state “Gaze perception involves both early visual processing (e.g., decoding the iris/pupil position relative to the sclera) and higher-level cognition (e.g., self-referential processing). When perceiving a gaze, visual signals—such as the contrast between the iris and the sclera—are first processed through the sensory system. These sensory inputs are then integrated with stored knowledge and self-referential expectations to form a holistic representation of whether or not others are looking at me [22,23].”

Citation: Langton, S. R. H. (2000). The mutual influence of gaze and head orientation in the analysis of social attention direction. *The Quarterly Journal of Experimental Psychology*, *53*(3), 825–845. <https://doi.org/10.1080/713755908>

Mareschal, I., Calder, A. J., Dadds, M. R., & Clifford, C. W. G. (2013). Gaze categorization under uncertainty: Psychophysics and modeling. *Journal of Vision*, *13*(5), 18. <https://doi.org/10.1167/13.5.18>

**Pg4 lines 73-93 – Methods of the studies reviewed could be summarised. In its current form, not clear how precision was operationalised (e.g., line 77).**

Thank you for these helpful suggestions. We have revised the second paragraph of the Introduction to include more details about the methods, now stating “Altered gaze perception has not only been reported in psychosis, ASD, and social anxiety disorder [12,18,34], but also in individuals with subclinical traits related to these disorders. *For instance, using the Cone of Direct Gaze (CoDG)—the range of gaze angles that one reports as receiving eye contact [25]—individuals with higher levels of social anxiety or psychosis proneness have been found to endorse a wider CoDG, suggesting they perceived direct eye contact from a wider range of gaze directions [17,21,35]. Individuals who endorsed more autism traits have similarly shown altered gaze perception, including decreased sensitivity to direct gaze [19] and a greater reliance on featural information (e.g., the size of the eyes)—rather than configural information (e.g., distance between facial features)—to detect self-directed gaze [36].* These findings suggest that aberrant gaze perception is not an all-or-nothing phenomenon that appears only in clinical populations; rather, it can also be present in the general population and the degree of disruption likely scales with levels of psychopathology. Furthermore, aberrant gaze perception observed across various psychopathology dimensions may be differentially subject to alterations in early visual and higher-level cognitive processing.” Changes are italicized for emphasis.

**Worthwhile to include a description of the mental conditions (social anxiety, autism, psychosis) and subclinical traits in the introduction, and their link to eye gaze perception and how any differences in gaze perception influence social cognition and interactions. This would provide a clear rationale for the study and its importance.**

We thank the reviewer for this important suggestion. We have restructured and expanded the introduction to reflect these changes. We wish to clarify that in our study, gaze perception serves as a key component of social cognition, as it constitutes a fundamental process involved in receiving and processing social cues (Yager & Ehmann, 2006). Therefore, any disruption in gaze perception is a specific component of broader social cognitive deficits and contributes to poorer social functioning. We have revised the introduction to make this conceptual link more explicit. We hope this revision addresses the reviewer's concern. Additions are italicized for emphasis.

The introduction now states, “Eye gaze is an important source of social information [1]. Perceiving and interpreting gaze direction helps us infer others’ intentions, identify the targets of their attention, and predict future communication [2]. *As such, the ability to precisely and efficiently identify others’ gaze direction is critical for navigating day-to-day interactions and represents a core component of broader social cognition [3,4]. This ability, however, is altered across psychopathologies characterized by social dysfunction, including schizophrenia [5–7], autism spectrum disorder (ASD; [8–11]), social anxiety disorder [12–14], and in populations with subclinical traits of these disorders [15–17], implying that it may impact social cognition across multiple psychopathology dimensions and contribute to poorer social functioning.* Therefore, disentangling the underlying components of gaze perception has the potential to elucidate the mechanisms contributing to social dysfunction. In this study, we combined experimental manipulations of stimuli in a gaze perception task and psychopathology trait measures to investigate mechanisms of altered gaze processing across psychopathology dimensions.”

Citations: Yager, J. A., & Ehmann, T. S. (2006). Untangling Social Function and Social Cognition: A Review of Concepts and Measurement. Psychiatry, 69(1), 47–68. https://doi.org/10.1521/psyc.2006.69.1.47

**Definitions needed for key terminology i.e. self-referential processing, when observed, why, and why it matters + what higher-level cognition involves, why threshold is primarily about higher-order cognitive processing (pg 5 line 99).**

We thank the reviewer for this helpful comment. The self-referential processing refers to the ability to judge if a gaze is self-directed or not (“looking at me”). As we mentioned earlier, the self-referential process is particularly critical when the gaze signal is ambiguous, such as when co-present directional signals conflict with one another. This process is of particular interest, as it is impaired in patients with schizophrenia (Chan et al., Tso et al., 2012) and predicts social functioning (Lasagna et al., 2024).

The threshold likely depends on both higher-order cognitive processing and perceptual visual processing. However, we argue that the threshold parameter in the present paradigm primarily reflects higher-order cognitive processing. First, the threshold represents the point at which a participant judges gaze as being directed toward themselves, spanning a range of angles with weaker signal strengths that introduce ambiguity. Therefore, our paradigm requires participants to resolve ambiguous gaze signals, where higher-order processing is more likely to play a determining role. Second, participants were explicitly instructed to respond to “Looking at me or not,” a framing that directly invokes self-referential processing. This judgment is conscious and deliberate, requiring participants to integrate perceptual information with self-referential evaluation under conditions of ambiguity.

We have revised the introduction accordingly. The updated paragraph now reads, “*Threshold reflects the degree of deviation from direct gaze that is interpreted as self-referential half of the time. Given that participants must explicitly judge whether gaze is directed at themselves across a continuum of gaze deviations, rather than simply detect eye orientation, this parameter primarily implicates higher-order cognitive processing [10,39]*.”

Citations: Tso, I. F., Mui, M. L., Taylor, S. F., & Deldin, P. J. (2012). Eye-contact perception in schizophrenia: Relationship with symptoms and socioemotional functioning. Journal of Abnormal Psychology, 121(3), 616–627. https://doi.org/10.1037/a0026596

Chan, S. K. W., Liu, T., Wong, A. O. Y., Wong, G. H. Y., Hsiao, J., Hui, C. L. M., Chang, W. C., Lee, E. H. M., & Chen, E. Y. H. (2021). Self-referential gaze perception of patients with schizophrenia and its relationship with symptomatology and cognitive functions. Schizophrenia Research, 228, 288–294. https://doi.org/10.1016/j.schres.2020.12.034

Stein, T., Senju, A., Peelen, M. V., & Sterzer, P. (2011). Eye contact facilitates awareness of faces during interocular suppression. Cognition, 119(2), 307–311. <https://doi.org/10.1016/j.cognition.2011.01.008>

Lasagna, C. A., Tso, I. F., Blain, S. D., & Pleskac, T. J. (2024). Cognitive mechanisms of aberrant self-referential social perception in psychosis and bipolar disorder: Insights from computational modeling. Schizophrenia Bulletin. <https://doi.org/10.1093/schbul/sbae147>

**Lines 84-84 – why expect these associations? Background on these conditions (i.e., what the literature shows re: when to expect low-level vs high-level effects) would clarify for the reader why you predict these distinct relations. Not clear from the introduction why these hypotheses are generated. Introduction needs revision to provide rationale for these. For example, there’s high comorbidity between autism and social anxiety, so why expect high social anxiety to be associated with greater self-referential bias but not high autistic traits?**

Thank you for this comment on theoretical rationale. We have substantially revised the introduction to provide a more detailed background for each hypothesis. Below is a copy of our revised hypothesis section (page 5) for ease of review.

“*We hypothesized that psychopathology dimensions would show differential levels of disruptions in these two processes, such that: 1) social anxiety would be associated with greater self-referential bias, in line with the wider CoDG reported in individuals with higher social anxiety [16,17,35,40], indicating a tendency to judge a broader range of gaze directions as self-directed; 2) autism would be associated with lower perceptual precision, as individuals with autism are less precise in detecting subtle changes in gaze angle [41]; and 3) psychosis proneness would be associated with lower perceptual precision and higher self-referential bias, consistent with prior findings linking psychosis proneness to wider CoDG and reduced precision [21,37].*”

**Gaze perception task pg 8 – If there’s a reason for having a fixed order of condition presentation across participants, it could be stated here. Otherwise, possible confounding effects of not having the order randomised.**

Thank you for asking for our rationale. A fixed order design was used to allow participants to progress through the gaze perception task with increasing difficulty (no noise, low noise, then high noise). This provided participants with sufficient trials to become acquainted with the task before encountering progressively ambiguous stimuli, with the aim of maintaining engagement and reducing frustrations that may occur if participants encountered the most challenging conditions first. Additionally, since the main focus of this work is individual differences, we implemented a fixed order to minimize potential variability in performance outcomes that could arise from differential order effects across participants, which would confound the analyses.

However, we acknowledge that a fixed order design limits our ability to make strong inferences about the influence of our experimental manipulations at the group level. We have added this caveat to the Limitation section: “Second, present findings suggest that adding visual noise *with a fixed order design* to gaze stimuli reduces perceptual precision and increases self-referential bias, yet conclusions about the impact on specific cognitive processes remain limited. Decreased perceptual precision could reflect disrupted early visual processing or changes in social cognition-specific processes. Likewise, enhanced self-referential bias could be caused by stronger reliance on prior beliefs or by strengthened perceptual biases [10]. *Future work needs to carefully rule out the practice or fatigue effects and better distinguish among these alternative explanations through computational modeling and/or neuroimaging*.”

**Clear description of the Methodology and reporting of Results. Minor comment: In Results, Table 2 shows results beyond what’s stated in the sentence that refers the reader to it.**

Thank you for noting this point. We have added a sentence to clarify what is included in Table 2 (Page 12, Line 260-261), stating “*Standardized path coefficients for associations between psychopathology traits and gaze task performance are provided in Table 2*.”

**It may also be worthwhile to more clearly highlight the individual-differences approach of the study (as opposed to clinical, with clinical diagnoses for SA, ASD, Schz.), as this is a real strength. Given the growing view of mental health as dimensional rather than categorical, emphasising this perspective would further strengthen the contribution of the work.**

We thank the reviewer for highlighting this strength of the manuscript and for the inspiring suggestion. We have taken this opportunity to more explicitly highlight this in the revised discussion. Below are the changes pertinent to this issue:

Discussion: “*Notwithstanding these limitations, the paper is the first to use an individual-differences approach to systematically compare shared and distinct mechanisms in gaze perception, and their associations with psychosis proneness, social anxiety, and autism traits in a community sample. This individual-differences approach has the benefit of investigating these clinical and behavioral phenotypes dimensionally, which is in line with empirical data supporting the dimensional conceptualization of psychopathology.*”

**Discussion: It may be helpful to begin the Discussion with a brief summary of all of the findings, explicitly noting which hypotheses were supported and which were not. This would help orient the reader and make the Discussion easier to follow.**

We thank the reviewer for this feedback. We added a brief summary paragraph to the beginning of the discussion, which states “*The current study combined a psychophysical gaze perception task with experimental stimulus manipulations, self-reports of psychopathology traits, and structural equation modeling to examine components of gaze perception and their disruption. Experimental manipulations of sensory quality (i.e., introducing gaussian noise over the stimuli) and context congruity (i.e., deviated-head orientation) successfully reduced perceptual precision and increased self-referential bias, respectively. Supporting our overarching hypothesis, our findings revealed that distinct psychopathology dimensions were associated with differential disruptions in gaze perception: autism traits were associated with lower perceptual precision, while higher social anxiety was associated with greater precision. However, not all predicted associations were supported, and the directions of some effects differed from our initial expectations. Specific hypotheses and interpretations are discussed, below, organized first around the basic experimental effects and then their associations with psychopathology traits*.”

**The findings for autistic traits are particularly compelling but are discussed only briefly. Higher autistic traits were associated with lower perceptual precision and also with greater self-referential bias. These results seem informative about gaze processing along the autism trait continuum, yet the Discussion does not fully explore their implications. For example, does reduced precision reflect differences in attention to the eye region, or greater perceptual uncertainty more generally? And how might increased self-referential bias relate to everyday social interpretation or ambiguity? You might also consider whether these findings fit within predictive processing or hypo-prior accounts of autistic perception, where weaker or less precise priors could lead to increased uncertainty under noise and downstream biases in social inference. Also, it would be important to be careful not to conflate higher autistic traits in the general population with a clinical diagnosis of ASD, and to make that distinction explicit in the Discussion.**

We thank the reviewer for noting the importance of these findings and for providing these valuable suggestions that could significantly strengthen our discussion. We have revised the discussion accordingly to detail the theoretical implications. In addition, we have added language to explicitly distinguish the higher autistic traits in the general population versus the clinical diagnosis of ASD. On page 16, it states, “Autism traits were associated with lower precision as hypothesized; this aligns with worse precision exhibited in ASD when discriminating subtle changes in gaze angle [12,41,69]. *This pattern may reflect a broader perceptual uncertainty in individuals with higher autism traits, rather than reduced attention to the eye region. While ASD and higher autism traits are often associated with gaze avoidance, recent eye-tracking evidence from a transdiagnostic sample (including participants spanning neurotypical to subclinical autism traits to ASD) using the same gaze perception task [70] indicated that the relationship between autism traits and reduced dwell time on the eyes emerges only after gaze direction judgements were made. This argues against a simple attentional account for worse precision among those high in autism traits.* Subjects with more autism traits also exhibited higher self-referential bias, suggesting they overestimated averted gaze as self-directed. Though this finding contradicts the result of Matsuyoshi et al. [36]—autism traits negatively correlated with self-referential bias—Dratsch et al. [71] suggested such inconsistencies may emerge from differences in the range of gaze angles examined. Elevated self-referential bias in ASD was specific to subtly averted gaze angles (1º-7º), whereas their performance was similar to controls for broader gaze-angle ranges (15º-25º; [71]). Further support is found in studies that examined gaze perception with stringent ranges (e.g., 0º-15º in [63]; 0º-10º in [72]). *Taken together, these findings dovetail with the hypo-prior accounts of perceptual experience in ASD [73], which postulate that weaker or less precise priors lead to a reduced capacity to resolve ambiguity through top-down processes, resulting in increased uncertainty and downstream biases in social inference. Future studies are needed to examine whether these findings generalize to individuals with a clinical diagnosis of ASD.*” Additions are italicized for emphasis.

**The findings for social anxiety traits (greater precision when perceiving changes in gaze angle) - The explanation (“They might discern ambiguous gaze and/or increased task difficulty as a threat, thereby initiating gaze avoidance which impedes perceptual precision.”) in terms of attentional bias and gaze avoidance is plausible, but as it stands it’s a bit hard to square with the finding that higher social anxiety was linked to better baseline precision; it doesn’t explain the higher baseline precision in high SA group. If gaze avoidance were the main mechanism, wouldn’t one expect poorer precision even when faces are clear? Could it be that socially anxious individuals closely monitor gaze when cues are clear, but become more uncertain or disengage (looking away as a regulation strategy?) as gaze information becomes noisier?**

We thank the reviewer for this insightful comment and for identifying a logical flaw in our original explanation. In response, we have revised our discussion to better account for the full pattern of findings. We now propose an explanation that considers the dynamic nature of attentional bias as a function of threat value, consistent with the “vigilance-avoidance” pattern of attentional bias (Mogg & Bradely, 1998, Koster et al., 2006).

We reorganized the paragraph the reviewer is referring to (now page 17) and added our speculation. Specifically: “Despite positive associations with overall precision, social anxiety was related to stronger negative effects of sensory noise on precision. In other words, although subjects with high social anxiety initially showed superior precision, this was attenuated after degrading the stimuli’s sensory quality. *This pattern may reflect dynamic attentional biases in anxious individuals that shift as a function of perceived threat value [79,80]. Heightened vigilance enabled them to closely monitor gaze cues, resulting in superior precision. However, when sensory noise increased ambiguity and task difficulty, socially anxious individuals may have interpreted the degraded stimuli as more threatening [81,82], instigating attentional avoidance that impeded perceptual precision [80].*” We hope this reframing offers a richer and more internally consistent explanation of our findings.

Citations: Koster, E. H. W., Crombez, G., Verschuere, B., Van Damme, S., & Wiersema, J. R. (2006). Components of attentional bias to threat in high trait anxiety: Facilitated engagement, impaired disengagement, and attentional avoidance. Behaviour Research and Therapy, 44(12), 1757–1771. <https://doi.org/10.1016/j.brat.2005.12.011>

Mogg, K., & Bradley, B. P. (1998). A cognitive-motivational analysis of anxiety. Behaviour Research and Therapy, 36(9), 809–848. https://doi.org/10.1016/S0005-7967(98)00063-1

**Paragraph on pg17 lines 366-374 is a bit confusing, as psychosis proneness was not related to precision or self-referential bias in the current data. The link here made to schizophrenia findings seems to imply a similar pattern that was not observed here.**

Thank you for highlighting this issue, and we agree with the reviewer that further clarifications are needed. Our results did not show a significant association between psychosis proneness and gaze perception metrics. The reference to schizophrenia findings was not intended to imply that a similar pattern was observed here, but rather to situate our null finding within a broader theoretical context. We believe the absence of an association in the current study may be due to the non-linear relationship between psychosis proneness and aberrant gaze perception across the psychosis continuum. Disruptions in precision and self-referential processing may only become detectable at higher levels of symptom severity, such as in clinical high-risk populations or individuals with schizophrenia-spectrum disorders. Our non-clinical sample is comprised of relatively healthy individuals, with low endorsement of perception anomalies and delusional beliefs, and are therefore not related to reduced precision or increased self-referential bias. This is consistent with previous findings in emotion processing (Fiorito et al., 2023) and cognitive deficits (Pilon, Giguère, & Potvin, 2025), suggesting that a strictly linear dimensional model may be insufficient to capture the full pattern of deficits across the psychosis spectrum (Ross & Margolis, 2019).

We have revised the paragraph to clarify this, “Against our hypotheses, psychosis proneness was not associated with low precision or high self-referential bias, contrasting with Chan et al.'s [52] findings of a positive association between psychosis symptom severity and self-referential bias in clinical samples. *Nonetheless, our null results may relate to the limited expressions of psychopathology in this relatively healthy sample—especially for psychosis proneness (Table 1, Fig S3)— and the possibly non-linear nature of this relationship across the psychosis continuum. Moreover, given findings and theories that negative symptoms and related traits (i.e., detachment) may be particularly connected to alterations in social cognition [73,86]—whereas the CAPS and PDI are conceptually related to positive symptoms—follow-up research should incorporate dimensional measures of social anhedonia and intimacy avoidance*.”

Citations: Fiorito, A. M., Aleman, A., Blasi, G., Bourque, J., Cao, H., Chan, R. C. K., Chowdury, A., Conrod, P., Diwadkar, V. A., Goghari, V. M., Guinjoan, S., Gur, R. E., Gur, R. C., Kwon, J. S., Lieslehto, J., Lukow, P. B., Meyer-Lindenberg, A., Modinos, G., Quarto, T., … Sescousse, G. (2023). Are Brain Responses to Emotion a Reliable Endophenotype of Schizophrenia? An Image-Based Functional Magnetic Resonance Imaging Meta-analysis. Biological Psychiatry, Schizophrenia: Novel Molecular and Circuit Mechanisms, 93(2), 167–177. https://doi.org/10.1016/j.biopsych.2022.06.013

Pilon, F., Giguère, C.-É., & Potvin, S. (2025). Rethinking the psychosis spectrum: A meta-analysis unveils a nonlinear discontinuity in verbal learning deficits between at-risk conditions and psychotic disorders. European Psychiatry, 68(1), e122. <https://doi.org/10.1192/j.eurpsy.2025.10094>

Ross, C. A., & Margolis, R. L. (2019). Research Domain Criteria: Strengths, Weaknesses, and Potential Alternatives for Future Psychiatric Research. Complex Psychiatry, 5(4), 218–236. https://doi.org/10.1159/000501797

**It may be helpful to include a brief paragraph in the Discussion outlining key study limitations. For example I wonder if COVID period (data collected in 2020) could have influenced participants’ self-report on questionnaires and/or gaze-processing.**

We thank the reviewer for this helpful suggestion. We have added a paragraph in the Discussion to specifically outline key study limitations (Page 18-19, Line 407-). It now states, “A few limitations are worth noting. First, our non-clinical sample reported a restricted range of psychopathology, particularly for psychosis proneness, which may have limited observable relations with gaze perception. The relatively modest sample size may also have affected the stability of parameter estimates and reduced statistical power. Future studies should recruit large transdiagnostic samples of patients with pronounced social deficits and those with subclinical levels of traits, as proposed by Tso et al. [88]. This could further validate these relations and potentially reveal both transdiagnostic and disorder-specific treatment targets for gaze processing deficits and associated social dysfunction. *Furthermore, participants completed the study during the COVID-19 pandemic, a period associated with elevated psychological stress. Although psychopathology trait scores in this sample were broadly comparable to those reported in pre-COVID studies conducted in the general population, it is possible that pandemic-related stressors may have heightened the levels of self-reported psychopathology traits [89], particularly for social anxiety [90] and the elevated autism traits [49,61].* Second, present findings suggest that adding visual noise with a fixed order design to gaze stimuli reduces perceptual precision and increases self-referential bias, yet conclusions about the impact on specific cognitive processes remain limited. Decreased perceptual precision could reflect disrupted early visual processing or changes in social cognition-specific processes. Likewise, enhanced self-referential bias could be caused by stronger reliance on prior beliefs or by strengthened perceptual biases [6]. Future work needs to carefully rule out the practice or fatigue effects and better distinguish among these alternative explanations through computational modeling and/or neuroimaging. Third, given the forced yes/no experimental design, the upper and lower asymptotes (i.e., lapse and guess rates) of the fitted logistic function were fixed when estimating gaze perception metrics. Future studies should consider occasional response errors and individual differences in subjective criteria for perceived self-directed gaze, particularly under noisier conditions, by allowing these asymptotes to vary and evaluating alternative lapse and guess rate specifications. Notwithstanding these limitations, the paper is the first to use an individual-differences approach to systematically compare shared and distinct mechanisms in gaze perception, and their associations with psychosis proneness, social anxiety, and autism traits in a community sample. This individual-differences approach has the benefit of investigating these clinical and behavioral phenotypes dimensionally, which is in line with empirical data supporting the dimensional conceptualization of psychopathology.” Additions are italicized for emphasis.

**Reviewer 2: Comments to authors:**

**Thanks for the opportunity to review this paper. Previous research has found that gaze perception can differ in association with traits of autism, schizotypy and social anxiety. The current study assesses all three psychological traits in the same sample to help tease apart similarities/differences in their influence on gaze perception. I think this is an interesting topic. I have a number of mostly-minor suggestions for the authors to consider.**

We thank the reviewer for acknowledging the value of this work and for their helpful suggestions. We have carefully revised the manuscript to address the noted concerns and incorporate the suggested revisions. The point-to-point responses are detailed below.

- **It seems important to the aims of the study that the collected sample captured sufficient variability in each of the psychological traits. I appreciate the descriptive statistics provided in Table 1 and Figure S3. I’d suggest commenting on this further in the paper. e.g., how do the descriptive statistics in your sample compare with the range/SD of scores found in larger samples in previous studies? (e.g., there are studies of the AQ with >1000 participants that might be a useful reference). How does the distribution of traits in your sample compare to any available cutoffs for these scales that suggest potentially clinically-relevant levels of these traits?**

We thank the reviewer for their valuable suggestions. We agree that providing broader context for the psychological trait variability in our sample would strengthen the manuscript. Overall, the current sample demonstrated comparable variability of the psychopathology traits relative to larger samples reported in previous studies, with slightly higher autism traits and trait asocial anxiety, and slightly lower trait psychosis proneness.

Regarding *autism traits*, the current study employed the 4-point Likert scoring for the AQ (range 50-200), resulting in a mean total score of 115.9 (14.9). This is slightly higher than, but still comparable to, the Dutch general population (*M_totalAQ_* = 104.20, *SD_totalAQ_* = 11.29, N = 302; Hoekstra et al., 2008) and the U.S. undergraduate sample (*M_totalAQ_* = 109.16, *SD_totalAQ_* = 12.91, N = 403; Stevenson & Hart, 2017). Since most of the literature that examined autism traits in the general population employ the traditional dichotomous scoring (“agree/disagree”, resulting in a total score ranging from 0-50), we also re-scored our data for the purpose of comparisons. The mean AQ-50 score was 20.42 (SD = 6.91, Range = 2-39), slightly higher than the mean of 16.94 (range 11.6 to 20.0) estimated from a systematic review of 6,924 nonclinical participants (Ruzich et al., 2015). The distribution of AQ scores in our sample (Figure S3) was also consistent with those reported in Ruzich et al. (2015). While the AQ is not a diagnostic tool, using the cut-off score of 32 or above suggested by Baron-Cohen et al. (2006), two participants may meet criteria of a potential diagnosis of ASD.

For *social anxiety*, the SAD-D scores (*M* = 10.9, *SD* = 7.7) were highly comparable to those reported in a larger Australian general population sample (Rice et al., 2021, *N* = 997, *M* = 10.8, *SD* = 8.89). On the other hand, the SPIN scores in our sample (*M* = 22.9, *SD* = 13.6) were higher than those reported in an adolescent sample (Ranta et al., 2007; *N* = 752, *M* = 11.3, *SD* =8.1) and healthy controls (Connor et al., 2018; *N* = 68, *M* =12.1, *SD* = 9.3). Notably, 50.5% of participants scored above the cut-off score of 20, which is indicative of mild social anxiety.

For the *psychosis proneness* trait, our sample endorsed fewer anomalous experiences and delusional beliefs compared to other studies. Specifically, our sample endorsed less anomalous perception experiences for CAPS (*M* = 5.4, *SD* = 5.2, Range = 0-27) than other non-clinical samples (*M* = 7.41, *SD* = 6.24, *N* = 193 in Bell et al., 2011; *M* = 7.3, *SD* = 5.8, Range = 0-26, *N* = 337 in Bell, Halligan, & Ellis, 2005), though intrusiveness, frequency, and distress scores were comparable. A similar pattern is illustrated in PDI, where the endorsed delusional beliefs (*M* = 4.5, *SD* = 3.4 Range = 0-14) are slightly lower than other non-clinical populations (*M* = 5.4, *SD* = 3.4, Range = 0-19, *N* = 337 in Bell, Halligan, & Ellis, 2005).

We have commented on it further in the discussion section in the manuscript, stating “*The distributions of psychopathology traits in our sample were broadly consistent with those reported in larger non-clinical samples, with mean levels of autism [53,65] and social anxiety [49,66,67] traits being comparably a bit higher, and slightly lower mean psychosis proneness [68]*.”

Citations: Hoekstra, R. A., Bartels, M., Cath, D. C., & Boomsma, D. I. (2008). Factor Structure, Reliability and Criterion Validity of the Autism-Spectrum Quotient (AQ): A Study in Dutch Population and Patient Groups. Journal of Autism and Developmental Disorders, 38(8), 1555–1566. <https://doi.org/10.1007/s10803-008-0538-x>

Stevenson, J. L., & Hart, K. R. (2017). Psychometric Properties of the Autism-Spectrum Quotient for Assessing Low and High Levels of Autistic Traits in College Students. Journal of Autism and Developmental Disorders, 47(6), 1838–1853. <https://doi.org/10.1007/s10803-017-3109-1>

Ruzich, E., Allison, C., Smith, P., Watson, P., Auyeung, B., Ring, H., & Baron-Cohen, S. (2015). Measuring autistic traits in the general population: A systematic review of the Autism-Spectrum Quotient (AQ) in a nonclinical population sample of 6,900 typical adult males and females. Molecular Autism, 6, 2. <https://doi.org/10.1186/2040-2392-6-2>

Baron-Cohen, S., Hoekstra, R. A., Knickmeyer, R., & Wheelwright, S. (2006). The Autism-Spectrum Quotient (AQ)—Adolescent Version. Journal of Autism and Developmental Disorders, 36(3), 343–350. https://doi.org/10.1007/s10803-006-0073-6

Connor, K. M., Davidson, J. R. T., Churchill, L. E., Sherwood, A., Weisler, R. H., & Foa, E. (2000). Psychometric properties of the Social Phobia Inventory (SPIN): New self-rating scale. British Journal of Psychiatry, 176(4), 379–386. <https://doi.org/10.1192/bjp.176.4.379>

Ranta, K., Kaltiala-Heino, R., Rantanen, P., Tuomisto, M. T., & Marttunen, M. (2007). Screening social phobia in adolescents from general population: The validity of the Social Phobia Inventory (SPIN) against a clinical interview. European Psychiatry, 22(4), 244–251. <https://doi.org/10.1016/j.eurpsy.2006.12.002>

Rice, K., Schutte, N. S., Rock, A. J., & Murray, C. V. (2021). Structure, validity and cut-off scores for the APA emerging measure: DSM-5 social anxiety disorder severity scale (SAD-D). Journal of Depression and Anxiety, 10(406), 2167–1044.

Bell, V., Halligan, P. W., Pugh, K., & Freeman, D. (2011). Correlates of perceptual distortions in clinical and non-clinical populations using the Cardiff Anomalous Perceptions Scale (CAPS): Associations with anxiety and depression and a re-validation using a representative population sample. Psychiatry Research, 189(3), 451–457. <https://doi.org/10.1016/j.psychres.2011.05.025>

Bell, V., Halligan, P. W., & Ellis, H. D. (2006). The Cardiff Anomalous Perceptions Scale (CAPS): A New Validated Measure of Anomalous Perceptual Experience. Schizophrenia Bulletin, 32(2), 366–377. https://doi.org/10.1093/schbul/sbj014

- **Relatedly, Figure S3 suggests a fairly normal distribution of scores for the AQ measures, while some of the other measures show very skewed distributions. Could this contribute to the results, in particular the lack of association found between the schizotypy measures and gaze perception, if your analyses are based on correlational techniques that assume normality?**

We thank the reviewer for pointing out this important observation. Unlike the AQ which was normally distributed, the distributions of trait psychosis proneness measures (CAPS and PDI) were right-skewed, consistent with patterns typically found in community and non-clinical samples (Bell et al., 2011; Bell et al., 2006). The majority of participants endorse very few anomalous perceptions or delusions, reflecting the nature of psychosis continuum. Thus, the low endorsement rates and restricted range of scores in our sample may have limited our ability to detect associations between psychosis proneness and gaze perception (as noted in “Limitations and Future Research”, pg. 18). Particularly, when taking into account the potential non-linear relationship between psychosis proneness and gaze perception, where meaningful disruptions in gaze perception may only emerge at subclinical or higher levels of psychosis proneness (e.g., ultra-high risk individuals for psychosis).

Regarding the potential effect of non-normality measures on structural equation modeling, we acknowledge this as a limitation and have added a note in the revised manuscript. At the same time, Jobst, Auersdwald, and Moshagen (2021) demonstrated that robust maximum likelihood estimation (which is employed in the current study) is relatively resilient to the violations of normality, showing only a slight effect on loading estimates and expected outcomes.

Citations: Bell, V., Halligan, P. W., Pugh, K., & Freeman, D. (2011). Correlates of perceptual distortions in clinical and non-clinical populations using the Cardiff Anomalous Perceptions Scale (CAPS): Associations with anxiety and depression and a re-validation using a representative population sample. Psychiatry Research, 189(3), 451–457. <https://doi.org/10.1016/j.psychres.2011.05.025>

Bell, V., Halligan, P. W., & Ellis, H. D. (2006). The Cardiff Anomalous Perceptions Scale (CAPS): A New Validated Measure of Anomalous Perceptual Experience. Schizophrenia Bulletin, 32(2), 366–377. https://doi.org/10.1093/schbul/sbj014

Jobst, L. J., Auerswald, M., & Moshagen, M. (2022). The Effect of Latent and Error Non-Normality on Measures of Fit in Structural Equation Modeling. Educational and Psychological Measurement, 82(5), 911–937. <https://doi.org/10.1177/00131644211046201>

- **I found the discussion of the effect of head orientation a bit unclear. Past research has found a net repulsive effect of head orientation on perceived gaze direction in naturalistic viewing conditions (e.g., Anstis et al 1969, Gamer & Hecht, 2007; Otsuka et al., 2015). In other words, a head rotated towards the left will tend to be perceived as looking slightly more rightwards than it really is, and vice versa. Figure 3-4 of the following paper provides a nice visualisation of this effect in the context of "looking at me" judgements: http://www.journalofvision.org/content/15/1/21 In the context of the current study (which only includes face stimuli for which head orientation is averted in the same direction as the averted eye directions), the repulsive effect of head orientation would render slightly-averted gaze being perceived as “looking at me”, while actually-direct gaze would be perceived as looking in the opposite direction to head rotation. This predicts that the threshold would shift towards averted gaze (as occurs in Figure 2) but also that the peak of the function may be lower if the repulsive effect is sufficient to push veridically-direct gaze in the direction opposite head orientation to a large enough extent that is not judged as looking at the observer. I imagine that the repulsive effect of head orientation on perceived gaze direction accounts for the differences in both threshold and width seen in Figure 2 for the “deviated” condition, but the way that the data are visualised (and the lack of stimuli with head/eyes averted in opposite directions) obscures this a little. It may be worth discussing the repulsive effect of head orientation in the Intro/Discussion and relating it to your findings, and/or providing some argument for any other effects that you think might be at play here.**
- **Page 5, line 115. The second hypothesis seems a bit weak or unclear to me. In line with my comment above regarding the repulsive effect of head orientation on gaze perception, I’d suggest that a weaker effect of head orientation on gaze perception would more likely reflect something like weaker holistic processing of the face / perceptual integration of face features. There are previous papers that discuss this in the context of gaze perception and autism, such as the following:** [**https://doi.org/10.1371/journal.pone.0275281**](https://doi.org/10.1371/journal.pone.0275281) [**https://pmc.ncbi.nlm.nih.gov/articles/PMC6942234/**](https://pmc.ncbi.nlm.nih.gov/articles/PMC6942234/)

We thank the reviewer for their sophisticated feedback regarding head orientation effects. Upon careful review of this literature, we found that head orientation manipulations have produced mixed findings (Moors et al., 2016; Kluttz et al., 2009). In addition to the repulsive effect suggested by the reviewer (“the perceived gaze direction is slightly biased in the opposite direction to the head rotation”, p.1, Otsuka, Mareschal, Clifford; 2015), several other distinct and sometimes contradictory effects have been reported, including the head-turn effect, the overestimation effect, the towing effect, and the Wollaston illusion. Below is a table that summarizes the definition of each.

| Head Orientation Effect | Operationalization |
| --- | --- |
| Head-turn Effect/ Repulsive Effect | The gaze direction of a human is perceived to be opposite to the head-turn. |
| Overestimation Effect | The perceived angle of the gaze is larger than the actual angle of the eye. |
| Towing Effect | The perceived direction of gaze is between the head and eye orientation. |
| Wollaston Illusion | Head orientation affects the perceived gaze direction even when the eye shape is identical in two different head orientations. |

A key contributor to this inconsistent literature is the considerable variations in how researchers have operationalized and measured head orientation effects, as illustrated in the table below.

| Study | Head Orientation Manipulation | Task / Response Format | Effect Found |
| --- | --- | --- | --- |
| Gibson & Pick (1963) | Front, 30° left, 30° right | Yes/No: "You are looking directly at me" | Head-turn Effect |
| Cline (1967) | 30° congruent vs. incongruent head-eye | Indicate perceived gaze direction on a response board. Each judgment was scored for its errors in vertical and horizontal cm. | Towing effect |
| Langton et al. (2004) | Congruent vs. incongruent; left/right & up/down | Indicate perceived gaze direction (left/right and up/down) | Observers were better at discriminating against gaze direction when the head and the gaze were congruent. |
| Conty et al. (2006) | Frontal vs. deviated head | Visual search detection task: detect whether a target gaze direction was present or not | 1. When the head was deviated, direct gaze was detected faster and better than an averted gaze.  2. Detection of averted gaze under deviated head yielded the slowest and worst performance. |
| Kluttz et al. (2009) | Front vs. turned; congruent vs. incongruent eyes | Judge where the model is looking at using a fixation bead along an optical bench rail. | Gaze in turned head was judged differently from gaze in straight head. |
| Otsuka et al. (2014) | Front, 30° left, 30° right | Judge the direction of gaze (averted left/direct/averted right) | Wollaston illusion |

The figure from Moors et al. (2016) further illustrates how the overshoot and towing effects manifest across gaze angles, underscoring that no single mechanism cleanly characterizes the literature.

(Moors et al., 2016)

Given these fundamental differences exist in how research questions are framed and how head orientations are measured, we felt it would be misleading to attribute our results to any single mechanism. Instead, in the Introduction, we described head orientation’s influence at a higher level, consistent with the broader pattern of evidence that head orientation robustly biases gaze judgements. Nevertheless, we agree that the repulsive effect may contribute to the present findings, and it is important to address it in the discussion. Therefore, we have expanded the Discussion to more thoroughly discuss the specific mechanisms that may underlie our findings, including the repulsive effect, and how it may account for the observed increased self-referential bias (threshold) and decreased precision (width) in the deviated head condition.

It now states, “We did not hypothesize that higher-level cognitive manipulations (i.e., the deviated head condition) would reduce precision, but this is also consistent with prior research [2,12]. This may emerge from incongruity between information sources (e.g., eyes and head). Although gaze can signal an individual’s target of attention, observers tend to perceive the face as a whole and exploit multiple sources of information when making judgments [23,56]. When context incongruity arises, it may disrupt participants’ certainty about their judgments, resulting in decreased precision. *The repulsive effect of head orientation may further contribute to this pattern, whereby a head rotated 30º to the left biases perceived gaze direction rightward (e.g., a true gaze of 25º left may be perceived as ~20º left) [21,57,58]. As a result, slightly averted gaze may be more likely to be judge as “looking at me”, whereas truly direct gaze may be perceived as slightly averted, thereby increasing self-referential bias and reducing precision.*” Changes are italicized for emphasis.

Citations: Moors, P., Verfaillie, K., Daems, T., Pomianowska, I., & Germeys, F. (2016). The Effect of Head Orientation on Perceived Gaze Direction: Revisiting Gibson and Pick (1963) and Cline (1967). Frontiers in Psychology, 7. <https://doi.org/10.3389/fpsyg.2016.01191>

Kluttz, N. L., Mayes, B. R., West, R. W., & Kerby, D. S. (2009). The effect of head turn on the perception of gaze. Vision Research, 49(15), 1979–1993. <https://doi.org/10.1016/j.visres.2009.05.013>

Gibson, J. J., & Pick, A. D. (1963). Perception of another person’s looking behavior. *The American Journal of Psychology*, *76*(3), 386. <https://doi.org/10.2307/1419779>

Cline, M. G. (1967). The perception of where a person is looking. The American Journal of Psychology, 80(1), 41. https://doi.org/10.2307/1420539

Langton, S. R. H., Honeyman, H., & Tessler, E. (2004). The influence of head contour and nose angle on the perception of eye-gaze direction. Perception & Psychophysics, 66(5), 752–771. <https://doi.org/10.3758/BF03194970>

Conty, L., Tijus, C., Hugueville, L., Coelho, E., & George, N. (2006). Searching for asymmetries in the detection of gaze contact versus averted gaze under different head views: A behavioural study. Spatial Vision, 19(6), 529–545. <https://doi.org/10.1163/156856806779194026>

Otsuka, Y., Mareschal, I., Calder, A. J., & Clifford, C. W. G. (2014). Dual-route model of the effect of head orientation on perceived gaze direction. Journal of Experimental Psychology: Human Perception and Performance, 40(4), 1425–1439. https://doi.org/10.1037/a0036151

- **For the gaze perception metrics (page 9, figure 1C), a logistic function is fit to the data and two parameters related the shape of this function (threshold and width) are used to quantify different aspects of gaze perception. Did the form of the logistic function also allow the upper bound of the function to vary or was this fixed to 1 as illustrated in Figure 1C? If the upper bound was fixed to 1, could variation in the peak of the data (e.g., the proportion of “looking at me” responses for the direct-gaze condition being lower on average for some noise/head conditions or varying across participants in relation to psychological traits) have a systematic effect on the estimated width/threshold parameters? If so, it might be worth checking that the same results come out when estimating the width/threshold parameters while allowing the upper bound of the logistic function to vary.**

We thank the reviewer for this careful observation. Our task utilized a forced Yes/No experiment design which required the participants to give a response to every trial to proceed. Since a response must be given to each trial and the response time was unlimited, our original model fixed the “lapse rate” (the difference between the upper asymptote and 1) and “guess rate” (the difference between the lower asymptote and 0) at 0, such that the upper and lower asymptotes are 1 and 0, respectively.

However, we acknowledge that even with this design, it is still possible for individuals to occasionally press the wrong (unintended) button or have different subjective criteria of perceived self-directed gaze. Thus, the actual upper and lower bounds of the data might not be perfectly 1 and 0, especially under noisier conditions. To test whether the selected upper and lower bounds have influence on our results, we refitted the gaze perception data by allowing 2 percent of “lapse rate” and “guess rate” as recommended by Kingdom and Prins (2010), essentially changing the lower and upper asymptotes to 0.02 and 0.98, respectively. As shown in the table below, the two sets of estimated gaze metrics were extremely highly correlated (r > 0.97 for all gaze metrics, with the majority exceeding r > 0.99; see table below), suggesting that the fixed upper bound in the original model did not significantly change the gaze parameter estimations. We therefore retained the original mode parameters for all reported analyses. Nevertheless, we acknowledge this as a methodological caveat and have added a note in our limitation section encouraging future studies to consider allowing these bounds to vary and test with different settings of lapse and guess rate.

| Gaze Parameter | Condition | *r* | *p* |
| --- | --- | --- | --- |
| Width | Forward No Noise | 0.990 | < .001 |
|  | Deviated No Noise | 0.970 | < .001 |
|  | Forward Low Noise | 0.995 | < .001 |
|  | Forward High Noise | 0.996 | < .001 |
| Threshold | Forward No Noise | 0.999 | < .001 |
|  | Deviated No Noise | 0.997 | < .001 |
|  | Forward Low Noise | 1.000 | < .001 |
|  | Forward High Noise | 1.000 | < .001 |

Limitation: “*Third, given the forced Yes/No experimental design, the upper and lower asymptotes (i.e., lapse and guess rates) of the fitted logistic function were fixed when estimating gaze perception metrics. Future studies should consider occasional response errors and individual differences in subjective criteria for perceived self-directed gaze, particularly under noisier conditions, by allowing these asymptotes to vary and evaluating alternative lapse and guess rate specifications.*”

Citations: Kingdom, F.A.A., Prins, N., 2010. Psychophysics: A practical introduction. Elsevier Academic Press, San Diego, CA, US

- **Also, am I correct in thinking that the width parameter is effectively the same as the slope/steepness of the logistic function? If not, how should one think of the difference between these?**

We thank the reviewer for this insightful question. The reviewer is correct that slope and width are conceptually related, as both indicate an individual’s sensitivity towards a gaze signal (indexing *precision*). However, they differ mathematically in important ways. The slope refers to the rate of change of the function at a specific point, typically measured when the endorsement rate is 50%. In earlier studies, we did use the slope to index the *perceptual precision,* with higher values indicating better sensitivity to weaker gaze signals. In contrast, the width of the function measures the difference between gaze angles at which eye contact is endorsed 5% and 95% of the time, and indexes the *perceptual imprecision*, with smaller values indicating more sensitivity to small changes in gaze angles. Importantly, width is expressed in the same unit as the threshold, making it easier to interpret than slope (Schütt et al., 2016). Furthermore, width retains the same meaning across all sigmoid function families, facilitating easy comparison across studies (Schütt et al., 2016). These additional clarifications have been added to the Supporting Information (Page 5, 1.3 Estimating Gaze Perception Metrics (Threshold, Width)), stating “The parameter *w* represents the width of the function, an inversion of slope for the psychometric function, which gauges the difference between gaze angles at which eye contact is endorsed 5% and 95% of the time. Compared to the slope parameter used in previous studies (e.g., in Lasagna et al., 2020), width has the practical advantage of being expressed in the same units as the threshold, making it easier to interpret. Furthermore, the width parameter holds consistent meaning across the family of sigmoid functions, facilitating easier comparisons across studies (Schütt et al., 2016).”

In addition to these conceptual advantages, our test-retest reliability findings (in Supporting Information S1.1.6) have demonstrated that width yielded higher reliability than slope estimates reported in our previous work (Lasagna et al., 2020), further strengthening arguments that favor width versus slop parameterizations of psychophysical logistic curves (Wichmann & Jäkel, 2018).

Citations: Lasagna, C. A., McLaughlin, M. M., Deng, W. Y., Whiting, E. L., & Tso, I. F. (2020). Deconstructing eye contact perception: Measuring perceptual precision and self-referential tendency using an online psychophysical eye contact detection task. *PLoS ONE*, *15*(3). <https://doi.org/10.1371/journal.pone.0230258>

Wichmann, F. A., & Jäkel, F. (2018). Methods in Psychophysics. In J. T. Wixted (Ed.), *Stevens’ Handbook of Experimental Psychology and Cognitive Neuroscience* (1st ed., pp. 1–42). Wiley. <https://doi.org/10.1002/9781119170174.epcn507>

Schütt, H. H., Harmeling, S., Macke, J. H., & Wichmann, F. A. (2016). Painfree and accurate Bayesian estimation of psychometric functions for (potentially) overdispersed data. *Vision Research*, *122*, 105–123. <https://doi.org/10.1016/j.visres.2016.02.002>

- **Page 5, line 99. “…related primarily to higher-order cognitive processing.” Is there anything that the authors can add here to substantiate the idea that the threshold parameter depends on higher-order cognitive processing, rather than being an aspect of visual processing? I think it is fine for the authors to apply their own interpretation, but I can also imagine the categorisation of gaze as being self-directed or not occurring at a more perceptual/sensory level. e.g., the findings by Timo Stein, Phillip Sterzer and colleagues that faces with direct gaze break suppression faster than faces with averted gaze might speak to the latter?**

We thank the reviewer for this helpful comment. Reviewer 1 raised a similar concern, and we agree that the threshold likely depends on both higher-order cognitive processing and perceptual visual processing. The work by Stein et al. (2013) elegantly demonstrated that eye contact information could be extracted automatically and robustly, reflecting that processing has happened at the early stages of processing. Recent computation work from our lab (Lasagna et al., 2026) has also shown that self-referential gaze processing involves both perceptual biases and belief-driven expectation bias.

However, we argue that the threshold parameter in the present paradigm primarily reflects higher-order cognitive processing. First, the threshold represents the point at which participants judge gaze as being directed toward themselves, spanning a range of angles with weaker signal strengths that introduce ambiguity. Unlike the clear gaze stimuli that has strong signal strength (direct gaze, averted gaze) in the Stein et al. (2013), our paradigm requires participants to resolve ambiguous gaze signals, and with this higher-order processing is more likely to play a determining role. Second, participants were explicitly instructed to respond to “Looking at me or not,” a framing that directly invokes self-referential processing. This judgment is both conscious and deliberate, requiring participants to integrate perceptual information with self-referential evaluation under conditions of ambiguity.

We have revised the introduction accordingly. The updated paragraph now reads, “Threshold reflects the degree of deviation from direct gaze that is interpreted as self-referential half of the time. Given that participants must explicitly judge whether gaze is directed at themselves across a continuum of gaze deviations, rather than simply detect eye orientation, this parameter primarily implicates higher-order cognitive processing [10,39].”

Citations: Stein, T., Senju, A., Peelen, M. V., & Sterzer, P. (2011). Eye contact facilitates awareness of faces during interocular suppression. Cognition, 119(2), 307–311. <https://doi.org/10.1016/j.cognition.2011.01.008>

Lasagna, C. A., Tso, I. F., Blain, S. D., & Pleskac, T. J. (2024). Cognitive mechanisms of aberrant self-referential social perception in psychosis and bipolar disorder: Insights from computational modeling. Schizophrenia Bulletin. <https://doi.org/10.1093/schbul/sbae147>

- **Figure 1 caption. The “Laterality” factor mentioned here isn’t explained in the Methods section.**

Thank you for this feedback. We have added the explanation to the Methods section. On page 9, Line 268, we state, “Nine incremental gaze angles were created by morphing realistic grayscale images from George et al. [44], consisting of six actors (three male, three female), using the “Abrosoft Fanta Morph” software (Beijing, China; see details in supplementary materials). Stimuli consisted of six actors (three male, three female) depicted in two head orientations, forward faces (with eyes averted left or right) and deviated faces (with head averted left or right).”

- **Page 9. Stimuli.**
- **Can you state the size of the face stimuli in visual angle, or at least give some indicative size in centimetres or range of sizes if the display settings were recorded for each participant.**

We thank the reviewer for this insightful methodological comment. We have presented the size of the stimuli on Page 4 of Supporting Information (the face stimuli are approximately 14.5cm tall and 10.8cm wide). Although individual display settings varied across participants given the online nature of the study, we implemented a novel pre-task calibration procedure to standardize the size of visual stimuli across participants. Specifically, participants were instructed to locate a credit card (an object found in most households with a globally standardized size) and resize their browser by zooming in or out until a credit card image on the screen matched their physical credit card. This procedure ensured that the face stimuli were displayed at approximately 14.5cm tall and 10.8 cm wide on the screen, regardless of the physical size of the monitor being used and provided a reasonable degree of control over the visual angle of the stimuli. Full details on the calibration procedure are provided in Supplemental Information S1.1.2.

- **When describing the parameters of the white noise, is the variance expressed in pixels? If so, it would be useful to note the size of the face images in pixels as well.**

We thank the reviewer for asking for clarification here. We used the “imnoise” function from MATLAB’s Image Processing Toolbox (R2019a) to add Gaussian white noise to the stimuli. The variance parameter (e.g., .01 in low noise condition) refers to Gaussian noise applied to pixel intensity values rather than spatial units.

- **The different gaze directions are created by morphing between faces with 0 and 10 degrees averted gaze. Does this really result in faces with a known angle of gaze? (e.g., 1.25 degrees etc as labelled in the figure). Or would it be more correct to express the different gaze directions in terms of % morph between the two original images? Perhaps including a little more detail here about the morphing technique would be useful.**

Thank you for raising this important point. The stimuli for different gaze directions were created by morphing the original photographs (0º and 30º) from George et al. (2001). Our previous study (Lasagna et al., 2020) has used a set of 11 images for each actor spanning from 0º and 30º in 3º increments. The 9 intermediate images for both forward and deviated faces were created using “Abrosoft Fanta Morph” software (Beijing, China) by morphing the two original images (0º and 30º) in 10% increments. In the present study, we selected a subset of the stimuli and created new gaze stimuli for forward faces (0º to 10º) and deviated faces (0º and 24º). The gaze angles (e.g., 1.25º averted) correspond to proportional increments of morphing between direct eye contact (0º) to most averted eyes (e.g., 10º in forward faces) rather than independently calibrated physical gaze angles.

We have clarified this description and added more details regarding the morphing technique in Supporting Information 1.1.3, stating “The original images from Geroge et al. (2001) were comprised of realistic grayscale photographs of six actors depicting two gaze directions: direct eye contact (0º; actor looking straight into the camera,) and 30º averted eye contact (actor looking 30º to the right of the camera). These photographs were also taken in two head orientations (forward, 30º deviated). To generate gaze stimuli spanning a continuous range of gaze angles, we followed the morphing protocol described in our previous work (Lasagna et al., 2020). In that study, nine intermediate images per actor were created between the two original images (0º and 30º) using 10% morph increments (“Abrosoft Fanta Morph” software; Beijing, China), yielding a set of 11 images with eyes averted leftward for 0º, 3º, 6º, 9º, 12º, 15º, 18º, 21º, 24º, 27º, and 30º. Each image was horizontally mirrored to produce a matched image of rightward-averted gaze stimuli. For the present study, we selected a subset of these morphed images to create nine stimuli spanning from 0º (“looking at me”) to 24º (“looking away from me”) in 3º increments for deviated faces (see Fig 1). The same process was applied to the forward-facing images, generating stimuli spanning 0º to 10º of gaze aversion for all actors.”

Citations: Lasagna, C. A., McLaughlin, M. M., Deng, W. Y., Whiting, E. L., & Tso, I. F. (2020). Deconstructing eye contact perception: Measuring perceptual precision and self-referential tendency using an online psychophysical eye contact detection task. PLoS ONE, 15(3). https://doi.org/10.1371/journal.pone.0230258

- **How was the sample size determined?**

We thank the reviewer for this important question. Determining an appropriate sample size for the present study was challenging, as the expected effect sizes for our primary hypotheses were difficult to determine. The existing literature on gaze perception mainly focuses on measuring the cone of direct gaze and rarely assesses the precision (width) and self-referential bias (threshold), making it difficult to empirically determine effect sizes for formal power analysis. Furthermore, existing gaze perception studies examining the effect of uncertainty using a psychophysical approach have typically recruited smaller samples. For instance, Mareschal et al. (2013) examined how gaze categorization changes under uncertainty (by adding noise to the eyes or removing face information) with only 9 observers. Similarly, their subsequent study investigated the effect of head orientation on gaze categorization with 12 observers (Mareschal, Otsuka, Clifford, 2014). In studies examining gaze perception across populations with subclinical psychopathology, relatively modest sample sizes were also employed, including those examining autistic traits (e.g., *N* = 20 in Madipakkam et al., 2018), trait social anxiety (e.g., *N* = 93 in Jun et al., 2013), and trait schizotypy (e.g., *N* = 62 in Wastler and Lenzenweger, 2018). In light of these considerations, we chose to recruit 30 participants per age/sex group, for a planned sample size of 120, expecting that this would allow us to detect the hypothesized effects.

We acknowledge that this approach carries limitations with respect to statistical power, and we have revised and expanded the Limitation section to address this concern more thoroughly, “A few limitations are worth noting. First, our non-clinical sample reported a restricted range of psychopathology, particularly for psychosis proneness, which may have limited observable relations with gaze perception. *The relatively modest sample size may also have affected the stability of parameter estimates and reduced statistical power. Future studies should recruit large transdiagnostic samples of patients with pronounced social deficits and those with subclinical levels of traits, as proposed by Tso et al. [88]. This could further validate these relations and potentially reveal both transdiagnostic and disorder-specific treatment targets for gaze processing deficits and associated social dysfunction.*”

Despite the limitation of the small sample size, this paper is the first to use an individual difference approach to systematically compare the shared and distinct mechanisms in gaze perception, and their associations with psychosis proneness, social anxiety, and autism traits in a community sample.

Citations: Mareschal, I., Otsuka, Y., & Clifford, C. W. G. (2014). A generalized tendency toward direct gaze with uncertainty. Journal of Vision, 14(12), 27. <https://doi.org/10.1167/14.12.27>

Mareschal, I., Calder, A. J., Dadds, M. R., & Clifford, C. W. G. (2013). Gaze categorization under uncertainty: Psychophysics and modeling. Journal of Vision, 13(5), 18. <https://doi.org/10.1167/13.5.18>

Madipakkam, A. R., Rothkirch, M., Dziobek, I., & Sterzer, P. (2019). Access to awareness of direct gaze is related to autistic traits. Psychological Medicine, 49(6), 980–986. https://doi.org/10.1017/S0033291718001630

Jun, Y. Y., Mareschal, I., Clifford, C. W. G., & Dadds, M. R. (2013). Cone of direct gaze as a marker of social anxiety in males. Psychiatry Research, 210(1), 193–198. <https://doi.org/10.1016/j.psychres.2013.05.020>

Wastler, H. M., & Lenzenweger, M. F. (2018). Cone of gaze in positive schizotypy: Relationship to referential thinking and social functioning. Personality Disorders, 9(4), 324–332. https://doi.org/10.1037/per0000258

- **Page 6. The number of participants recruited stated here differs slightly from the Abstract.**

Thank you for noting us about this discrepancy, and we apologize for any confusion that this may have caused. Initially, the number reported in the Abstract reflected the total number of participants recruited prior to data quality screening, whereas the number reported on Page 6 reflected the final analysis sample following exclusions. To resolve this discrepancy, we have updated the Abstract to report the final analysis sample (*N* = 106), which now states “A general population sample of 106 participants completed an online psychophysical gaze perception task and a battery of psychopathology traits questionnaires.
