# Supplementary material for: Gaze processing across psychosis proneness, social anxiety, and autism traits: Examining the role of sensory quality and context congruity
Source: PLoS One. 2026 Sep 3;21(9):e0352116. doi: 10.1371/journal.pone.0352116 (PMC13541124; doi:10.1371/journal.pone.0352116)
Supplement: Supplementary file 4 [file pone.0352116.s005.docx]

I think the **Introduction and Discussion** need major revisions.

**Introduction** pg3 paragraph 2 – what constitutes prior information in the context of gaze perception not clear.

Pg4 lines 73-93 – Methods of the studies reviewed could be summarised. In its current form, not clear how precision was operationalised (e.g., line 77).

Worthwhile to include a description of the mental conditions (social anxiety, autism, psychosis) and subclinical traits in the introduction, and their link to eye gaze perception and how any differences in gaze perception influence social cognition and interactions. This would provide a clear rationale for the study and its importance.

Definitions needed for key terminology i.e. **self-referential processing**, when observed, why, and why it matters + what **higher-level cognition** involves, why threshold is primarily about higher-order cognitive processing (pg 5 line 99).

Lines 84-84 – why expect these associations? Background on these conditions (i.e., what the literature shows re: when to expect low-level vs high-level effects) would clarify for the reader why you predict these distinct relations.

Not clear from the introduction why these hypotheses are generated. Introduction needs revision to provide rationale for these. For example, there’s high comorbidity between autism and social anxiety, so why expect high social anxiety to be associated with greater self-referential bias but not high autistic traits?

Gaze perception task pg 8 – If there’s a reason for having a fixed order of condition presentation across participants, it could be stated here. Otherwise, possible confounding effects of not having the order randomised.

Clear description of the Methodology and reporting of Results. Minor comment: In Results, Table 2 shows results beyond what’s stated in the sentence that refers the reader to it.

It may also be worthwhile to more clearly highlight the individual-differences approach of the study (as opposed to clinical, with clinical diagnoses for SA, ASD, Schz.), as this is a real strength. Given the growing view of mental health as dimensional rather than categorical, emphasising this perspective would further strengthen the contribution of the work.

**Discussion**

It may be helpful to begin the Discussion with a brief summary of all of the findings, explicitly noting which hypotheses were supported and which were not. This would help orient the reader and make the Discussion easier to follow.

The findings for autistic traits are particularly compelling but are discussed only briefly. Higher autistic traits were associated with lower perceptual precision and also with greater self-referential bias. These results seem informative about gaze processing along the autism trait continuum, yet the Discussion does not fully explore their implications. For example, does reduced precision reflect differences in attention to the eye region, or greater perceptual uncertainty more generally? And how might increased self-referential bias relate to everyday social interpretation or ambiguity? You might also consider whether these findings fit within predictive processing or hypo-prior accounts of autistic perception, where weaker or less precise priors could lead to increased uncertainty under noise and downstream biases in social inference. Also, it would be important to be careful not to conflate higher autistic traits in the general population with a clinical diagnosis of ASD, and to make that distinction explicit in the Discussion.

The findings for social anxiety traits (greater precision when perceiving changes in gaze angle) - The explanation (“They might discern ambiguous gaze and/or increased task difficulty as a threat, thereby initiating gaze avoidance which impedes perceptual precision.”) in terms of attentional bias and gaze avoidance is plausible, but as it stands it’s a bit hard to square with the finding that higher social anxiety was linked to better baseline precision; it doesn’t explain the higher baseline precision in high SA group. If gaze avoidance were the main mechanism, wouldn’t one expect poorer precision even when faces are clear? Could it be that socially anxious individuals closely monitor gaze when cues are clear, but become more uncertain or disengage (looking away as a regulation strategy?) as gaze information becomes noisier?

Paragraph on pg17 lines 366-374 is a bit confusing, as psychosis proneness was not related to precision or self-referential bias in the current data. The link here made to schizophrenia findings seems to imply a similar pattern that was not observed here.

It may be helpful to include a brief paragraph in the Discussion outlining key study limitations. For example I wonder if COVID period (data collected in 2020) could have influenced participants’ self-report on questionnaires and/or gaze-processing.
