# Supplementary material for: Gaze processing across psychosis proneness, social anxiety, and autism traits: Examining the role of sensory quality and context congruity
Source: PLoS One. 2026 Sep 3;21(9):e0352116. doi: 10.1371/journal.pone.0352116 (PMC13541124; doi:10.1371/journal.pone.0352116)
Supplement: Peer review history

# Peer review: Decision Letter 0

**DOI:** 10.1371/journal.pone.0352116.r001

**Authors:** Jeroen J. A. van Boxtel

24 Feb 2026

Dear Dr. Tso,

First let me apologise again for the delay in providing you with feedback. I have now finally secured two reviewers. They are generally quite positive about the manuscript, but have some requests about statistically adequacy, which  you will need to address. And they ask for several other clarifications, which I urge you to full address as well (please note that reviewer 1 has attached a file with additional comments. Let me know if you do not have access to that). Importantly, it appears that the data is not made fully and freely available. The statements provided indicate that some restrictions apply, but it is not explained why that is the case. I cannot accept the article without the data being made publically available (there are some rare exceptions, but a very clear ethical or legal reason needs to be provided in those cases). Thank you again for your patience, and I am looking forward to seeing an updated manuscript. Please see the standard letter below.

Please submit your revised manuscript by Apr 10 2026 11:59PM. If you will need more time than this to complete your revisions, please reply to this message or contact the journal office at plosone@plos.org. When you're ready to submit your revision, log on to https://www.editorialmanager.com/pone/ and select the 'Submissions Needing Revision' folder to locate your manuscript file.

- A letter that responds to each point raised by the academic editor and reviewer(s). You should upload this letter as a separate file labeled 'Response to Reviewers'.
- A marked-up copy of your manuscript that highlights changes made to the original version. You should upload this as a separate file labeled 'Revised Manuscript with Track Changes'.
- An unmarked version of your revised paper without tracked changes. You should upload this as a separate file labeled 'Manuscript'.

If applicable, we recommend that you deposit your laboratory protocols in protocols.io to enhance the reproducibility of your results. Protocols.io assigns your protocol its own identifier (DOI) so that it can be cited independently in the future. For instructions see: https://journals.plos.org/plosone/s/submission-guidelines#loc-laboratory-protocols. Additionally, PLOS ONE offers an option for publishing peer-reviewed Lab Protocol articles, which describe protocols hosted on protocols.io. Read more information on sharing protocols at https://plos.org/protocols?utm_medium=editorial-email&utm_source=authorletters&utm_campaign=protocols.

We look forward to receiving your revised manuscript.

Kind regards,

Jeroen J. A. van Boxtel

Academic Editor

PLOS One

Journal Requirements:

If the reviewer comments include a recommendation to cite specific previously published works, please review and evaluate these publications to determine whether they are relevant and should be cited. There is no requirement to cite these works unless the editor has indicated otherwise.

Please review your reference list to ensure that it is complete and correct. If you have cited papers that have been retracted, please include the rationale for doing so in the manuscript text, or remove these references and replace them with relevant current references. Any changes to the reference list should be mentioned in the rebuttal letter that accompanies your revised manuscript. If you need to cite a retracted article, indicate the article’s retracted status in the References list and also include a citation and full reference for the retraction notice.

[Note: HTML markup is below. Please do not edit.]

Reviewers' comments:

Reviewer's Responses to Questions

**Comments to the Author**

1. Is the manuscript technically sound, and do the data support the conclusions?

Reviewer #1: Partly

Reviewer #2: Yes

**********

2. Has the statistical analysis been performed appropriately and rigorously?

Reviewer #1: I Don't Know

Reviewer #2: Yes

**********

3. Have the authors made all data underlying the findings in their manuscript fully available?

The PLOS Data policy

Reviewer #1: No

Reviewer #2: No

**********

4. Is the manuscript presented in an intelligible fashion and written in standard English?

Reviewer #1: Yes

Reviewer #2: Yes

**********

Reviewer #1: Data for the study is not availabe. My understanding is that this is a requirement for PlosOne.

I'm not in a position to comment on the robustness of the statistical approach as I'm not familiar with ESEM, but I do have some questions regarding whether the sample size is sufficient for the individual-differences analyses conducted here. Further comments on the Introduction and the Discussion are uploaded as an attachment.

Reviewer #2: Thanks for the opportunity to review this paper. Previous research has found that gaze perception can differ in association with traits of autism, schizotypy and social anxiety. The current study assesses all three psychological traits in the same sample to help tease apart similarities/differences in their influence on gaze perception. I think this is an interesting topic. I have a number of mostly-minor suggestions for the authors to consider.

– It seems important to the aims of the study that the collected sample captured sufficient variability in each of the psychological traits. I appreciate the descriptive statistics provided in Table 1 and Figure S3. I’d suggest commenting on this further in the paper. e.g., how do the descriptive statistics in your sample compare with the range/SD of scores found in larger samples in previous studies? (e.g., there are studies of the AQ with >1000 participants that might be a useful reference). How does the distribution of traits in your sample compare to any available cutoffs for these scales that suggest potentially clinically-relevant levels of these traits?

– Relatedly, Figure S3 suggests a fairly normal distribution of scores for the AQ measures, while some of the other measures (e.g., PDI, CAPS) show very skewed distributions. Could this contribute to the results, in particular the lack of association found between the schizotypy measures and gaze perception, if your analyses are based on correlational techniques that assume normality?

– I found the discussion of the effect of head orientation a bit unclear. Past research has found a net repulsive effect of head orientation on perceived gaze direction in naturalistic viewing conditions (e.g., Anstis et al 1969, Gamer & Hecht, 2007; Otsuka et al., 2015). In other words, a head rotated towards the left will tend to be perceived as looking slightly more rightwards than it really is, and vice versa. Figure 3-4 of the following paper provides a nice visualisation of this effect in the context of "looking at me" judgements: http://www.journalofvision.org/content/15/1/21 In the context of the current study (which only includes face stimuli for which head orientation is averted in the same direction as the averted eye directions), the repulsive effect of head orientation would render slightly-averted gaze being perceived as “looking at me”, while actually-direct gaze would be perceived as looking in the opposite direction to head rotation. This predicts that the threshold would shift towards averted gaze (as occurs in Figure 2) but also that the peak of the function may be lower if the repulsive effect is sufficient to push veridically-direct gaze in the direction opposite head orientation to a large enough extent that is is not judged as looking at the observer. I imagine that the repulsive effect of head orientation on perceived gaze direction accounts for the differences in both threshold and width seen in Figure 2 for the “deviated” condition, but the way that the data are visualised (and the lack of stimuli with head/eyes averted in opposite directions) obscures this a little. It may be worth discussing the repulsive effect of head orientation in the Intro/Discussion and relating it to your findings, and/or providing some argument for any other effects that you think might be at play here.

– Page 5, line 115. The second hypothesis seems a bit weak or unclear to me. In line with my comment above regarding the repulsive effect of head orientation on gaze perception, I’d suggest that a weaker effect of head orientation on gaze perception would more likely reflect something like weaker holistic processing of the face / perceptual integration of face features. There are previous papers that discuss this in the context of gaze perception and autism, such as the following:

https://doi.org/10.1371/journal.pone.0275281

https://pmc.ncbi.nlm.nih.gov/articles/PMC6942234/

– For the gaze perception metrics (page 9, figure 1C), a logistic function is fit to the data and two parameters related the shape of this function (threshold and width) are used to quantify different aspects of gaze perception. Did the form of the logistic function also allow the upper bound of the function to vary or was this fixed to 1 as illustrated in Figure 1C? If the upper bound was fixed to 1, could variation in the peak of the data (e.g., the proportion of “looking at me” responses for the direct-gaze condition being lower on average for some noise/head conditions or varying across participants in relation to psychological traits) have a systematic effect on the estimated width/threshold parameters? If so, it might be worth checking that the same results come out when estimating the width/threshold parameters while allowing the upper bound of the logistic function to vary.

– Also, am I correct in thinking that the width parameter is effectively the same as the slope/steepness of the logistic function? If not, how should one think of the difference between these?

– Page 5, line 99. “…related primarily to higher-order cognitive processing.” Is there anything that the authors can add here to substantiate the idea that the threshold parameter depends on higher-order cognitive processing, rather than being an aspect of visual processing? I think it is fine for the authors to apply their own interpretation, but I can also imagine the categorisation of gaze as being self-directed or not occurring at a more perceptual/sensory level. e.g., the findings by Timo Stein, Phillip Sterzer and colleagues that faces with direct gaze break suppression faster than faces with averted gaze might speak to the latter?

– Figure 1 caption. The “Laterality” factor mentioned here isn’t explained in the Methods section.

– Page 9. Stimuli.

– Can you state the size of the face stimuli in visual angle, or at least give some indicative size in centimetres or range of sizes if the display settings were recorded for each participant.

– When describing the parameters of the white noise, is the variance expressed in pixels? If so, it would be useful to note the size of the face images in pixels as well.

– The different gaze directions are created by morphing between faces with 0 and 10 degrees averted gaze. Does this really result in faces with a known angle of gaze? (e.g., 1.25 degrees etc as labelled in the figure). Or would it be more correct to express the different gaze directions in terms of % morph between the two original images? Perhaps including a little more detail here about the morphing technique would be useful.

– How was the sample size determined?

– Page 6. The number of participants recruited stated here differs slightly from the Abstract.

**********

what does this mean?). If published, this will include your full peer review and any attached files.

If you choose “no”, your identity will remain anonymous but your review may still be made public.

**Do you want your identity to be public for this peer review?**  For information about this choice, including consent withdrawal, please see our Privacy Policy

Reviewer #1: **Yes:**  Özge Karakale

Reviewer #2: No

**********

[NOTE: If reviewer comments were submitted as an attachment file, they will be attached to this email and accessible via the submission site. Please log into your account, locate the manuscript record, and check for the action link "View Attachments". If this link does not appear, there are no attachment files.]

To ensure your figures meet our technical requirements, please review our figure guidelines: https://journals.plos.org/plosone/s/figures

You may also use PLOS’s free figure tool, NAAS, to help you prepare publication quality figures: https://journals.plos.org/plosone/s/figures#loc-tools-for-figure-preparation.

NAAS will assess whether your figures meet our technical requirements by comparing each figure against our figure specifications.

Attachment Submitted filename: Comments.docx

# Author comment: Author response to Decision Letter 1

**DOI:** 10.1371/journal.pone.0352116.r002

1 May 2026

Dear Dr. Jeroen J. A. van Boxtel,

Thank you very much for the opportunity to submit a revision of our manuscript, “Gaze Processing Across Psychosis Proneness, Social Anxiety, and Autism Traits: Examining the Role of Sensory Quality and Context Congruity.” We also thank the anonymous reviewers for their time, thoughtful comments, and guidance to improve the original submission. We have carefully revised the manuscript to address the noted concerns and incorporate the suggested revisions. Importantly, the de-identified dataset is now publicly available on OSF: https://osf.io/chqw2. We believe the manuscript has substantially improved. Below, we detail our responses to each of the reviewer’s comments (which are in bold). We sometimes repeat our responses for similar comments raised by multiple reviewers for ease of review. Thank you very much for your valuable time and effort in improving this manuscript.

Sincerely,

Pan Gu and co-authors

Editor’s Notes:

Reviewer 1: Comments to authors:

Data for the study is not available. My understanding is that this is a requirement for PlosOne. I'm not in a position to comment on the robustness of the statistical approach as I'm not familiar with ESEM, but I do have some questions regarding whether the sample size is sufficient for the individual-differences analyses conducted here. Further comments on the Introduction and the Discussion are uploaded as an attachment.

Thank you for raising this data availability concern. In response to this and the editor’s request, we have shared the dataset on OSF: https://osf.io/chqw2.

Regarding the sample size, we acknowledge the limitations of a relatively small sample size, and future studies are needed to replicate the current study in a bigger sample. We agree that larger samples are more powered for individual-difference analyses using SEM. However, we note that similar sample sizes have been used successfully in previous studies. For example, Schmidt, Mueller, and Roder (2011) conducted SEM to examine how social cognition mediates the relationship between neurocognitive performance and functional status among 148 schizophrenia patients.

We have also revised and expanded the Limitation section to more thoroughly address this concern, “A few limitations are worth noting. First, our non-clinical sample reported a restricted range of psychopathology, particularly for psychosis proneness, which may have limited observable relations with gaze perception. The relatively modest sample size may also have affected the stability of parameter estimates and reduced statistical power. Future studies should recruit large transdiagnostic samples of patients with pronounced social deficits and those with subclinical levels of traits, as proposed by Tso et al. [88]. This could further validate these relations and potentially reveal both transdiagnostic and disorder-specific treatment targets for gaze processing deficits and associated social dysfunction.”

Despite the limitation of a small sample size, this paper is the first to use an individual difference approach to systematically compare the shared and distinct mechanisms in gaze perception, and their associations with psychosis proneness, social anxiety, and autism traits in a community sample.

Citations: Schmidt, S. J., Mueller, D. R., & Roder, V. (2011). Social Cognition as a Mediator Variable Between Neurocognition and Functional Outcome in Schizophrenia: Empirical Review and New Results by Structural Equation Modeling. Schizophrenia Bulletin, 37(suppl_2), S41–S54. https://doi.org/10.1093/schbul/sbr079

I think the Introduction and Discussion need major revisions. Introduction pg3 paragraph 2 – what constitutes prior information in the context of gaze perception not clear.

We thank the reviewer for these valuable suggestions. We have carefully revised the manuscript to address the noted concerns regarding the introduction and discussion.

For Introduction paragraph 2 (Page 3), we thank the reviewer for raising this concern regarding the clarity of prior information in the context of gaze perception. Here, examples for higher-level cognition processes include self-referential processing, emotional expression, and attentional control. These top-down processes are particularly critical when co-present directional signals conflict with one another, such as when head orientation is incongruent with eye direction, or when pointing gestures are incongruent with eye direction (Langton, 2000). In such cases, observers draw on prior knowledge and expectations to resolve the ambiguous sources of information, form a holistic judgement of eye gaze, and most often to think others were “looking at me” (Mareschal et al., 2013). We have revised the paragraph to reflect these changes. Specifically, on page 3, we now state “Gaze perception involves both early visual processing (e.g., decoding the iris/pupil position relative to the sclera) and higher-level cognition (e.g., self-referential processing). When perceiving a gaze, visual signals—such as the contrast between the iris and the sclera—are first processed through the sensory system. These sensory inputs are then integrated with stored knowledge and self-referential expectations to form a holistic representation of whether or not others are looking at me [22,23].”

Citation: Langton, S. R. H. (2000). The mutual influence of gaze and head orientation in the analysis of social attention direction. The Quarterly Journal of Experimental Psychology, 53(3), 825–845. https://doi.org/10.1080/713755908

Mareschal, I., Calder, A. J., Dadds, M. R., & Clifford, C. W. G. (2013). Gaze categorization under uncertainty: Psychophysics and modeling. Journal of Vision, 13(5), 18. https://doi.org/10.1167/13.5.18

Pg4 lines 73-93 – Methods of the studies reviewed could be summarised. In its current form, not clear how precision was operationalised (e.g., line 77).

Thank you for these helpful suggestions. We have revised the second paragraph of the Introduction to include more details about the methods, now stating “Altered gaze perception has not only been reported in psychosis, ASD, and social anxiety disorder [12,18,34], but also in individuals with subclinical traits related to these disorders. For instance, using the Cone of Direct Gaze (CoDG)—the range of gaze angles that one reports as receiving eye contact [25]—individuals with higher levels of social anxiety or psychosis proneness have been found to endorse a wider CoDG, suggesting they perceived direct eye contact from a wider range of gaze directions [17,21,35]. Individuals who endorsed more autism traits have similarly shown altered gaze perception, including decreased sensitivity to direct gaze [19] and a greater reliance on featural information (e.g., the size of the eyes)—rather than configural information (e.g., distance between facial features)—to detect self-directed gaze [36]. These findings suggest that aberrant gaze perception is not an all-or-nothing phenomenon that appears only in clinical populations; rather, it can also be present in the general population and the degree of disruption likely scales with levels of psychopathology. Furthermore, aberrant gaze perception observed across various psychopathology dimensions may be differentially subject to alterations in early visual and higher-level cognitive processing.” Changes are italicized for emphasis.

Worthwhile to include a description of the mental conditions (social anxiety, autism, psychosis) and subclinical traits in the introduction, and their link to eye gaze perception and how any differences in gaze perception influence social cognition and interactions. This would provide a clear rationale for the study and its importance.

We thank the reviewer for this important suggestion. We have restructured and expanded the introduction to reflect these changes. We wish to clarify that in our study, gaze perception serves as a key component of social cognition, as it constitutes a fundamental process involved in receiving and processing social cues (Yager & Ehmann, 2006). Therefore, any disruption in gaze perception is a specific component of broader social cognitive deficits and contributes to poorer social functioning. We have revised the introduction to make this conceptual link more explicit. We hope this revision addresses the reviewer's concern. Additions are italicized for emphasis.

The introduction now states, “Eye gaze is an important source of social information [1]. Perceiving and interpreting gaze direction helps us infer others’ intentions, identify the targets of their attention, and predict future communication [2]. As such, the ability to precisely and efficiently identify others’ gaze direction is critical for navigating day-to-day interactions and represents a core component of broader social cognition [3,4]. This ability, however, is altered across psychopathologies characterized by social dysfunction, including schizophrenia [5–7], autism spectrum disorder (ASD; [8–11]), social anxiety disorder [12–14], and in populations with subclinical traits of these disorders [15–17], implying that it may impact social cognition across multiple psychopathology dimensions and contribute to poorer social functioning. Therefore, disentangling the underlying components of gaze perception has the potential to elucidate the mechanisms contributing to social dysfunction. In this study, we combined experimental manipulations of stimuli in a gaze perception task and psychopathology trait measures to investigate mechanisms of altered gaze processing across psychopathology dimensions.”

Citations: Yager, J. A., & Ehmann, T. S. (2006). Untangling Social Function and Social Cognition: A Review of Concepts and Measurement. Psychiatry, 69(1), 47–68. https://doi.org/10.1521/psyc.2006.69.1.47

Definitions needed for key terminology i.e. self-referential processing, when observed, why, and why it matters + what higher-level cognition involves, why threshold is primarily about higher-order cognitive processing (pg 5 line 99).

We thank the reviewer for this helpful comment. The self-referential processing refers to the ability to judge if a gaze is self-directed or not (“looking at me”). As we mentioned earlier, the self-referential process is particularly critical when the gaze signal is ambiguous, such as when co-present directional signals conflict with one another. This process is of particular interest, as it is impaired in patients with schizophrenia (Chan et al., Tso et al., 2012) and predicts social functioning (Lasagna et al., 2024).

The threshold likely depends on both higher-order cognitive processing and perceptual visual processing. However, we argue that the threshold parameter in the present paradigm primarily reflects higher-order cognitive processing. First, the threshold represents the point at which a participant judges gaze as being directed toward themselves, spanning a range of angles with weaker signal strengths that introduce ambiguity. Therefore, our paradigm requires participants to resolve ambiguous gaze signals, where higher-order processing is more likely to play a determining role. Second, participants were explicitly instructed to respond to “Looking at me or not,” a framing that directly invokes self-referential processing. This judgment is conscious and deliberate, requiring participants to integrate perceptual information with self-referential evaluation under conditions of ambiguity.

We have revised the introduction accordingly. The updated paragraph now reads, “Threshold reflects the degree of deviation from direct gaze that is interpreted as self-referential half of the time. Given that participants must explicitly judge whether gaze is directed at themselves across a continuum of gaze deviations, rather than simply detect eye orientation, this parameter primarily implicates higher-order cognitive processing [10,39].”

Citations: Tso, I. F., Mui, M. L., Taylor, S. F., & Deldin, P. J. (2012). Eye-contact perception in schizophrenia: Relationship with symptoms and socioemotional functioning. Journal of Abnormal Psychology, 121(3), 616–627. https://doi.org/10.1037/a0026596

Chan, S. K. W., Liu, T., Wong, A. O. Y., Wong, G. H. Y., Hsiao, J., Hui, C. L. M., Chang, W. C., Lee, E. H. M., & Chen, E. Y. H. (2021). Self-referential gaze perception of patients with schizophrenia and its relationship with symptomatology and cognitive functions. Schizophrenia Research, 228, 288–294. https://doi.org/10.1016/j.schres.2020.12.034

Stein, T., Senju, A., Peelen, M. V., & Sterzer, P. (2011). Eye contact facilitates awareness of faces during interocular suppression. Cognition, 119(2), 307–311. https://doi.org/10.1016/j.cognition.2011.01.008

Lasagna, C. A., Tso, I. F., Blain, S. D., & Pleskac, T. J. (2024). Cognitive mechanisms of aberrant self-referential social perception in psychosis and bipolar disorder: Insights from computational modeling. Schizophrenia Bulletin. https://doi.org/10.1093/schbul/sbae147

Lines 84-84 – why expect these associations? Background on these conditions (i.e., what the literature shows re: when to expect low-level vs high-level effects) would clarify for the reader why you predict these distinct relations. Not clear from the introduction why these hypotheses are generated. Introduction needs revision to provide rationale for these. For example, there’s high comorbidity between autism and social anxiety, so why expect high social anxiety to be associated with greater self-referential bias but not high autistic traits?

Thank you for this comment on theoretical rationale. We have substantially revised the introduction to provide a more detailed background for each hypothesis. Below is a copy of our revised hypothesis section (page 5) for ease of review.

“We hypothesized that psychopathology dimensions would show differential levels of disruptions in these two processes, such that: 1) social anxiety would be associated with greater self-referential bias, in line with the wider CoDG reported in individuals with higher social anxiety [16,17,35,40], indicating a tendency to judge a broader range of gaze directions as self-directed; 2) autism would be associated with lower perceptual precision, as individuals with autism are less precise in detecting subtle changes in gaze angle [41]; and 3) psychosis proneness would be associated with lower perceptual precision and higher self-referential bias, consistent with prior findings linking psychosis proneness to wider CoDG and reduced precision [21,37].”

Gaze perception task pg 8 – If there’s a reason for having a fixed order of condition presentation across participants, it could be stated here. Otherwise, possible confounding effects of not having the order randomised.

Thank you for asking for our rationale. A fixed order design was used to allow participants to progress through the gaze perception task with increasing difficulty (no noise, low noise, then high noise). This provided participants with sufficient trials to become acquainted with the task before encountering progressively ambiguous stimuli, with the aim of maintaining engagement and reducing frustrations that may occur if participants encountered the most challenging conditions first. Additionally, since the main focus of this work is individual differences, we implemented a fixed order to minimize potential variability in performance outcomes that could arise from differential order effects across participants, which would confound the analyses.

However, we acknowledge that a fixed order design limits our ability to make strong inferences about the influence of our experimental manipulations at the group level. We have added this caveat to the Limitation section: “Second, present findings suggest that adding visual noise with a fixed order design to gaze stimuli reduces perceptual precision and increases self-referential bias, yet conclusions about the impact on specific cognitive processes remain limited. Decreased perceptual precision could reflect disrupted early visual processing or changes in social cognition-specific processes. Likewise, enhanced self-referential bias could be caused by stronger reliance on prior beliefs or by strengthened perceptual biases [10]. Future work needs to carefully rule out the practice or f

Attachment Submitted filename: ResponseLetter_GazeNoise.docx

# Peer review: Decision Letter 1

**DOI:** 10.1371/journal.pone.0352116.r003

**Authors:** Jeroen J. A. van Boxtel

4 Jun 2026

Gaze Processing Across Psychosis Proneness, Social Anxiety, and Autism Traits: Examining the Role of Sensory Quality and Context Congruity

PONE-D-25-55945R1

Dear Dr. Tso,

We’re pleased to inform you that your manuscript has been judged scientifically suitable for publication and will be formally accepted for publication once it meets all outstanding technical requirements.

Within one week, you’ll receive an e-mail detailing the required amendments. When these have been addressed, you’ll receive a formal acceptance letter and your manuscript will be scheduled for publication.

An invoice will be generated when your article is formally accepted. Please note, if your institution has a publishing partnership with PLOS and your article meets the relevant criteria, all or part of your publication costs will be covered. Please make sure your user information is up-to-date by logging into Editorial Manager at Editorial Manager® and clicking the ‘Update My Information' link at the top of the page. For questions related to billing, please contact billing support.

If your institution or institutions have a press office, please notify them about your upcoming paper to help maximize its impact. If they’ll be preparing press materials, please inform our press team as soon as possible -- no later than 48 hours after receiving the formal acceptance. Your manuscript will remain under strict press embargo until 2 pm Eastern Time on the date of publication. For more information, please contact onepress@plos.org.

Kind regards,

Jeroen J. A. van Boxtel

Academic Editor

PLOS One

Additional Editor Comments (optional):

Congratulations, you have succesfully addressed the reviewers' concerns, and I am happy to accept the paper for publication.

Reviewers' comments:

Reviewer's Responses to Questions

**Comments to the Author**

Reviewer #1: All comments have been addressed

Reviewer #2: All comments have been addressed

**********

2. Is the manuscript technically sound, and do the data support the conclusions?

Reviewer #1: Yes

Reviewer #2: Yes

**********

3. Has the statistical analysis been performed appropriately and rigorously?

Reviewer #1: I Don't Know

Reviewer #2: Yes

**********

4. Have the authors made all data underlying the findings in their manuscript fully available?

The PLOS Data policy

Reviewer #1: Yes

Reviewer #2: Yes

**********

5. Is the manuscript presented in an intelligible fashion and written in standard English?

Reviewer #1: Yes

Reviewer #2: Yes

**********

Reviewer #1: Key points I raised have been addressed. The introduction and discussion sections have improved in terms of clarity of the hypotheses, rationale, level of explanation for key concepts/theories and interpretation provided for the results. Thank you.

Reviewer #2: Thanks to the authors for considering my comments. I have no further suggestions.

**********

what does this mean?). If published, this will include your full peer review and any attached files.

If you choose “no”, your identity will remain anonymous but your review may still be made public.

**Do you want your identity to be public for this peer review?**  For information about this choice, including consent withdrawal, please see our Privacy Policy

Reviewer #1: **Yes:**  Ozge Karakale

Reviewer #2: No

**********

# Editor report: Acceptance letter

**DOI:** 10.1371/journal.pone.0352116.r004

**Authors:** Jeroen J. A. van Boxtel

PONE-D-25-55945R1

PLOS One

Dear Dr. Tso,

I'm pleased to inform you that your manuscript has been deemed suitable for publication in PLOS One. Congratulations! Your manuscript is now being handed over to our production team.

At this stage, our production department will prepare your paper for publication. This includes ensuring the following:

* All references, tables, and figures are properly cited

* All relevant supporting information is included in the manuscript submission,

* There are no issues that prevent the paper from being properly typeset

You will receive further instructions from the production team, including instructions on how to review your proof when it is ready. Please keep in mind that we are working through a large volume of accepted articles, so please give us a few days to review your paper and let you know the next and final steps.

Lastly, if your institution or institutions have a press office, please let them know about your upcoming paper now to help maximize its impact. If they'll be preparing press materials, please inform our press team within the next 48 hours. Your manuscript will remain under strict press embargo until 2 pm Eastern Time on the date of publication. For more information, please contact onepress@plos.org.

You will receive an invoice from PLOS for your publication fee after your manuscript has reached the completed accept phase. If you receive an email requesting payment before acceptance or for any other service, this may be a phishing scheme. Learn how to identify phishing emails and protect your accounts at https://explore.plos.org/phishing.

If we can help with anything else, please email us at customercare@plos.org.

Thank you for submitting your work to PLOS One and supporting open access.

Kind regards,

PLOS ONE Editorial Office Staff

on behalf of

Dr. Jeroen J. A. van Boxtel

Academic Editor

PLOS One
